# Advances in Intra-Articular Injection Hydrogel Drug Delivery Systems in the Treatment of Rheumatoid Arthritis

**DOI:** 10.3390/pharmaceutics17091118

**Published:** 2025-08-27

**Authors:** Mong-Hsiu Song, Yuxuan Yan, Bohan Chen, Liming Gong, Liqing Chen, Jing Feng, Mingfeng Han, Chenfei Liu, Congcong Xiao, Mingji Jin, Zhonggao Gao, Wei Huang

**Affiliations:** 1 State Key Laboratory of Bioactive Substance and Function of Natural Medicines, Department of Pharmaceutics, Institute of Materia Medica, Chinese Academy of Medical Sciences and Peking Union Medical College, Beijing 100050, China; songjosh@imm.ac.cn (M.-H.S.); chenbohan@imm.ac.cn (B.C.); gongliming@imm.ac.cn (L.G.); chenliqing@imm.ac.cn (L.C.); fengjinga@imm.ac.cn (J.F.); liuchenfei@imm.ac.cn (C.L.); xiaocongcong@imm.ac.cn (C.X.); jinmingji@imm.ac.cn (M.J.); 2 Beijing Key Laboratory of Drug Delivery Technology and Novel Formulations, Department of Pharmaceutics, Institute of Materia Medica, Chinese Academy of Medical Sciences and Peking Union Medical College, Beijing 100050, China; 3 Department of Pharmacology, School of Pharmacy, Fourth Military Medical University, Xi’an 710032, China; seraphy0519@163.com; 4 School of Pharmacy, Yanbian University, Yanji 133002, China; 18698857919@163.com

**Keywords:** rheumatoid arthritis, hydrogel, intra-articular, multimodal therapy, combination therapy

## Abstract

Rheumatoid arthritis (RA) is a systemic autoimmune disorder characterized by chronic inflammation of the synovial membrane, leading to synovial hyperplasia, infiltration of immune cells, and subsequent cartilage and bone erosion. This progressive joint pathology results in persistent pain and functional impairment. Currently, convenient oral traditional disease-modifying anti-rheumatic drugs (DMARDs) are available, and increasingly precise biologic agents and targeted synthetic DMARDs (tsDMARDs) have been developed, offering promising therapeutic options. However, systemic administration generally fails to achieve therapeutic drug concentrations in the joints owing to poor biodistribution and dose-limiting systemic toxicity. Intra-articular (IA) administration has demonstrated promising potential in addressing these challenges. Among the various strategies employed for IA administration, hydrogels have gained significant attention due to their tunable mechanical properties, biocompatibility, and controlled release capabilities. These unique properties enable hydrogel-based IA delivery systems to simultaneously modulate the inflammatory microenvironment and protect cartilage tissue. This review comprehensively summarizes the histopathological changes and associated cellular and molecular events in RA, while also highlighting the design principles of hydrogels and advanced strategies for hydrogel-based IA administration. By addressing the limitations of conventional treatments, hydrogel-based IA injection holds significant promise for improving RA treatment.

## 1. Introduction

Rheumatoid arthritis (RA) is a long-term autoimmune condition that causes inflammation in the joints and surrounding soft tissues, leading to discomfort, swelling, and progressive damage to joint structures [1]. This autoimmune disorder typically affects small joints in a symmetrical pattern, particularly those in the hands and feet [2,3]. The 2017 Global Burden of Disease study reported that the global age-standardized incidence of RA rose by 8.2% (95% CI 5.9–10.5%) and its prevalence increased by 7.4% (95% CI 5.3–9.4%) between 1990 and 2017 [4]. RA patients have a significantly increased incidence of morbidity, disability, and mortality compared to the general population [2,3].

At the cellular level, the development of RA involves synovial hyperplasia, angiogenesis, and the infiltration of inflammatory cells [2]. Pro-inflammatory cytokines are released by activated T cells to mediate immune responses and enhance synovial inflammation [5]. Macrophages are crucial in the development of RA. They release cytokines to activate osteoclasts and contribute to both cartilage degradation and the proliferation of fibroblast-like synoviocytes (FLSs) [2,6]. Autoantibodies promote RA pathogenesis by forming immune complexes that cause inflammation and tissue damage [7]. Key signaling pathways, such as JAK-STAT and NF-κB, drive the persistent inflammatory cascade in RA [8]. Great challenges still remain for RA management due to the need for lifelong treatment to control disease progression [9,10].

Therapeutic agents for RA have advanced considerably over the last few decades. These agents mainly include disease-modifying anti-rheumatic drugs (DMARDs), targeted synthetic DMARDs (tsDMARDs), and biologic agents [11]. Their therapeutic actions are initiated through diverse mechanisms. DMARDs relieve RA progression by regulating the immune system and reducing joint inflammation, while tsDMARDs function by inhibiting the Janus kinase pathway to suppress cytokine-driven inflammation and modulate immune responses. Biologic agents exert therapeutic effects by targeting specific components of the immune system. However, these therapies are associated with increased risk of infections and variable patient responses [12,13]. Biologic agents and tsDMARDs are highly targeted to modulate immune responses but may cause hematologic and hepatic side effects [12]. Due to the accumulated substantial efficacy and safety data, methotrexate (MTX) still remains the primary treatment for RA even with its slow onset and safety issues [1,14,15,16,17]. These limitations suggest the need for developing more precise and effective delivery systems.

Intra-articular (IA) delivery system is a promising strategy for RA management. This system directly targets the affected joints and greatly reduces the toxicity caused by systemic drug circulation. However, frequent repeated injections are required due to the therapeutics either being absorbed by synovial tissues or degraded in synovial fluid within hours. Novel drug delivery systems have been constructed to overcome this problem, including both non-hydrogel systems (such as nanoparticles and microparticles) and hydrogel-based platforms. Compared to non-hydrogel systems, hydrogels demonstrate several distinct advantages. While nanoparticles and microparticles can extend drug residence time, hydrogels offer superior biocompatibility and better mimicry of native extracellular matrix (ECM) due to their three-dimensional (3D) hydrophilic polymer networks. This ECM-like structure not only provides better tissue integration but also enables more controlled drug release kinetics. Furthermore, hydrogels can be engineered with stimuli-responsive properties (e.g., responsive to pH, temperature, or enzymatic activity within inflamed joints) that are difficult to achieve with conventional nanoparticle systems [16,18].

However, each system has its limitations. Nanoparticles and microparticles may face challenges in terms of homogeneous distribution and potential migration from the injection site, while hydrogel-based therapies, despite their significant potential, may be affected by the dynamic inflammatory environment within the synovial cavity, which could hinder sustained drug release and compromise therapeutic efficacy [16]. The choice between these systems often depends on the specific therapeutic requirements, with hydrogels being particularly suitable for applications requiring prolonged residence time and microenvironment-responsive drug release.

This review provides a comprehensive overview of the pathophysiological mechanisms underlying RA, with a particular focus on the key cellular players and molecular pathways involved in disease progression. Unlike previous reviews, we systematically analyze how recent advances in understanding these mechanisms have directly informed the rational design of injectable hydrogels over the past five years. Specifically, we critically evaluate (1) RA-mediated cellular reprogramming within synovial tissue microenvironments, (2) the rational engineering principles for hydrogel-based therapeutics, and (3) the biomaterial innovations targeting these pathological cellular processes. By bridging fundamental mechanistic insights with translational hydrogel engineering, this review offers a unique perspective on developing precision biomaterials for RA management.

## 2. Histopathological Changes and Associated Cellular and Molecular Events in RA 

**Figure 1:**
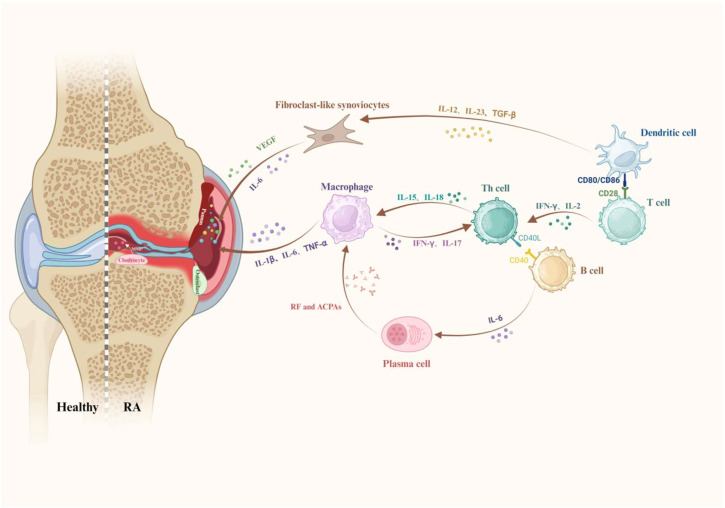
Pathogenic mechanisms of RA progression. Synergistic interactions between M1 macrophages (TNF-α/IL-1β), Th17 cells (IL-17), autoantibody-producing B cells, and activated fibroblasts (MMPs/RANKL) drive synovitis, cartilage erosion, and bone destruction. DCs present autoantigens, while osteoclasts mediate joint damage, creating self-perpetuating inflammation. Created using https://BioRender.com.

The joint is a complex structure composed of two or more bones interconnected by articular cartilage, which minimizes friction. It is surrounded by fibrous connective tissue and synovial membranes. Recent studies have revealed that RA pathogenesis involves multi-site injury of joint tissues, characterized by progressive angiogenesis, inflammation, and synovial hyperplasia. These pathological changes disrupt the joint microenvironment, causing heterogeneous infiltration of inflammatory cells, pathological changes in synovial and joint tissues, dysregulation of inflammatory mediators and signaling pathways, and severe reduction–oxidation (redox) imbalance in the synovium and surrounding joint tissues. Understanding the histopathological changes and the underlying cellular and molecular mechanisms is critical for developing targeted therapies for RA. The pathogenic mechanisms underlying RA inflammation are illustrated in Figure 1.

### 2.1. Heterogeneous Infiltration of Inflammatory Cells

During RA progression, the synovial tissue within affected joints undergoes pathological changes, characterized by heterogeneous infiltration of immune cells, including T cells, macrophages, B cells, neutrophils, and dendritic cells (DCs). These populations collectively sustain chronic inflammation through distinct mechanisms, ultimately driving the progressive joint destruction of RA [2].

#### 2.1.1. T Cells

T cells, particularly autoreactive CD4^+^ T helper (Th) cells, play a central role in the pathogenesis of RA, driving inflammation and joint destruction through their interactions with other immune cells and synovial tissues [19,20,21]. CD4^+^ T cells differentiate into subsets such as Th1, Th17, and regulatory T cells (Tregs), each uniquely contributing to disease progression [21,22]. Th1 and Th17 cells drive inflammation by secreting pro-inflammatory cytokines like IFN-γ and IL-17, which activate FLSs and macrophages, leading to the production of matrix metalloproteinases (MMPs) and other inflammatory mediators [23]. In contrast, Tregs are often functionally impaired in RA [24]. This disrupts the balance between pro-inflammatory and regulatory mechanisms. Among CD4^+^ T cells, T follicular helper (Tfh) cells promote B cell differentiation into antibody-producing plasma cells and memory B cells through cytokines like IL-21 [25,26]. The expanded Tfh cell population promotes the production of pathogenic autoantibodies [e.g., rheumatoid factor (RF) and anti-citrullinated protein antibodies (ACPAs)] [26]. The survival and proliferation of autoimmune B cells are also enhanced by Tfh cells. Such functional interactions between Tfh and B cells are mediated by co-stimulatory molecules such as ICOS/ICOSL and CD40/CD40L [26]. Additionally, Tfh-derived IL-21 promotes Th17 cell differentiation, creating a self-sustaining inflammatory loop [26].

#### 2.1.2. Monocytes and Macrophages

The pathogenesis of RA is critically driven by monocytes and macrophages [27,28]. Specifically, circulating monocytes are recruited to the synovial tissue in response to chemokines such as CXCL12 and CX3CL1 [27,29]. Following tissue infiltration, these monocytes differentiate into antigen-presenting DCs or pro-inflammatory macrophages, becoming key mediators of the inflammatory cascade [30]. Activated monocytes and their derivatives not only perpetuate synovial inflammation through the secretion of pro-inflammatory cytokines (such as TNF-α, IL-1β, and IL-6), but they also directly participate in the formation of pannus [29]. Moreover, these cells stimulate the release of MMPs and other degradative enzymes [31]. Meanwhile, monocytes contribute to irreversible joint damage through activating fibroblasts and osteoclasts [29,32].

Monocytes undergo significant phenotypic and functional shifts characterized by an enhanced pro-inflammatory capacity and upregulated surface markers [e.g., CD14, CD16, and Toll-like receptors (TLRs)] [27,30,33]. These shifts are driven by both intrinsic genetic factors and extrinsic environmental triggers, particularly pro-inflammatory cytokines and immune complexes [34]. They also promote oxidative stress via reactive oxygen species (ROS) overproduction [35].

Macrophages are exposed to persistent stimulation through high concentrations of pro-inflammatory cytokines (e.g., TNF-α and IL-1β) [28]. This sustained activation drives macrophages to generate tissue-destructive ROS and secrete proteolytic enzymes including MMPs. Beyond inducing abnormal activation of FLSs, these activated macrophages directly mediate the progressive destruction of articular matrix [36]. Furthermore, they contribute to disease progression through multiple mechanisms: (1) mediating chemotaxis and abnormal proliferation of synovial endothelial cells, promoting pathological pannus formation; (2) facilitating the infiltration of inflammatory cells; and (3) secreting pro-angiogenic factors including vascular endothelial growth factor (VEGF) and IL-8, which induce pathological angiogenesis [37]. Additionally, the pannus tissue formed under macrophage dominance exhibits invasive properties, leading to irreversible joint destruction [2].

#### 2.1.3. B Cells

During the pathogenesis of RA, B lymphocytes participate in disease progression through multiple mechanisms [38]. B cells activate T cells to initiate adaptive immune responses by serving as antigen-presenting cells (APCs) and sustain autoimmune responses via the persistent release of co-stimulatory signals. The pathogenic antibodies they produce directly contribute to inflammatory tissue injury in the synovium by forming immune complexes that activate the complement system [38]. Moreover, B cells secrete pro-inflammatory cytokines, VEGF, and macrophage migration inhibitory factor, leading to inflammation. Notably, B cells can differentiate into plasma cells that persistently produce autoantibodies, perpetuating chronic inflammation [39,40]. The presence and activity of these plasma cells in the synovium correlate with RA severity [39]. Additionally, the ectopic lymphoid structures formed in synovial tissues provide a microenvironment for long-term B cell survival, demonstrating their pivotal role in RA [41].

#### 2.1.4. Neutrophils

Neutrophils, as quintessential pro-inflammatory immune cells, actively recruit other immune cells in RA [42]. These innate immune cells are directly involved in immune responses. The survival of neutrophils is significantly prolonged following inflammatory activation, thereby exerting significant impacts on disease progression [43]. Neutrophils drive RA progression through various mechanisms, including the production of immune mediators [ROS, chemokines, and neutrophil extracellular traps (NETs)] via respiratory burst, and their prolonged survival due to impaired apoptosis [42,44]. NETs are extracellular DNA structures containing histones and neutrophil-derived proteins [45]. Activated neutrophils demonstrate enhanced NET formation, which exposes autoantigens and damage-associated molecular patterns, thereby triggering inflammatory responses [46,47]. These NETs contain citrullinated proteins, which activate FLSs in the affected joints. The citrullinated proteins are recognized by ACPAs and can trigger the formation of pathogenic immune complexes within the joints. 

#### 2.1.5. DCs

DCs significantly influence the pathogenesis of RA by migrating to inflamed synovial tissues, where they activate various immune cells through antigen presentation and cytokine production [32]. TLR engagement promotes metabolic switching in DCs from mitochondrial respiration to glycolytic metabolism, supporting their acute inflammatory and antigen-presenting capacities [35]. DCs critically control the activation and differentiation of Th17 cells, which are relevant to the induction and propagation of chronic inflammation in RA [48]. In ACPA-positive RA patients, an increased population of plasmacytoid dendritic cells (pDCs) has been observed [49]. These pDCs promote autoantibody production by expressing anti-apoptotic B cell-activating factor (BAFF), which enhances B cell survival and differentiation [50]. Additionally, synovial DCs express lower levels of CCR7, a chemokine receptor essential for their migration to lymph nodes. This reduced CCR7 expression leads to the retention of mature DCs within inflamed tissues, thereby maintaining inflammation [51].

### 2.2. Pathological Changes in Synovial and Joint Tissues

The synovium and joint tissues in RA undergo pathological changes characterized by three key processes: FLSs activation, pannus invasion, and metabolic dysfunction and oxidative stress. These interconnected mechanisms collectively contribute to the irreversible joint damage.

#### 2.2.1. Fibroblast-like Synoviocytes (FLSs)

FLSs experience a sequence of pathological changes during RA progression [29]. They acquire an activated phenotype through epigenetic reprogramming, leading to aggressive behaviors [29]. A recent study has shown that the pathogenic characteristics of FLSs persist even after prolonged culture in vitro or transplantation into mice, indicating that this transformation is independent of inflammatory stimuli [52]. FLSs secrete various pathogenic mediators (e.g., MMPs and other degradative enzymes) and exhibit enhanced migratory and invasive capabilities, which are maintained through epigenetic mechanisms that perpetuate their pathological phenotype [52,53,54]. Inflammation is sustained through their interactions with immune cells, while osteoclast activation is promoted, thereby accelerating joint damage [53,55]. Furthermore, through dual mechanisms involving type I interferon expression and GM-CSF secretion, FLSs prevent apoptosis of both T cells and neutrophils, delaying inflammation resolution [29].

#### 2.2.2. Pannus

A pannus is a hallmark histological feature of RA. It is a granulation tissue composed of proliferating synovial cells, infiltrating inflammatory cells, and newly formed blood vessels [56]. This pathological tissue invades the articular cartilage surface and causes erosion of cartilage and bone. The pannus can directly disrupt joint structure and secrete inflammatory cytokines and degradative enzymes [56]. FLS migration and invasion significantly contribute to pannus development [57]. Notably, angiogenesis occurs at an early stage of RA progression, with newly formed blood vessels supporting the sustained growth and invasiveness of the pannus [58,59]. Thus, an invasive pannus may extend into surrounding tissues without timely intervention [1]. The interplay of inflammatory mediators, immune dysregulation, hypoxic conditions, and angiogenic factors collectively drives pannus formation and disease progression [59].

#### 2.2.3. Metabolic Dysregulation and Oxidative Stress

The accumulation of metabolic waste, particularly lactic acid, reduces the pH of the joint cavity [60,61,62]. Synovial cells adapt to the pro-inflammatory microenvironment by changing their metabolic pathways. These adaptations enhance the invasiveness of synovial tissue within the RA joint [62]. The acidic microenvironment compromises chondrocyte anabolic activity, resulting in reduced ECM biosynthesis and concomitant enhancement of cartilage catabolism [63]. Notably, metabolic intermediates and enzymes involved in glycolysis may function as autoantigens [62]. 

Oxidative stress, characterized by an elevated production of ROS and diminished antioxidant defenses, represents a key hallmark of RA [62]. Within inflamed synovial tissue, macrophages, neutrophils, and other activated immune cells produce excessive ROS via NADPH oxidase activity and mitochondrial dysfunction [62]. Such oxidative stress destroys joints by inducing lipid peroxidation, protein oxidation, and DNA damage [64,65]. Moreover, ROS function as signaling molecules that activate the NF-κB and MAPK pathways [66]. The activation of these pathways upregulates the expression of pro-inflammatory cytokines, thereby maintaining synovial inflammation [66]. The depletion of antioxidants [glutathione, superoxide dismutase (SOD), and catalase (CAT)] disturbs redox balance. Such redox imbalances also influence the behavior of FLSs, which become resistant to apoptosis and adopt a hyperproliferative, invasive phenotype [62,67]. Additionally, oxidative stress modifies self-proteins, generating neoepitopes that trigger autoantibody production, further driving autoimmune responses in RA [68]. 

### 2.3. Inflammatory Mediators and Signaling Pathways 

RA progression involves a coordinated interplay of inflammatory mediators and dysregulated signaling pathways. Pro-inflammatory cytokines and chemokines drive synovitis through NF-κB and JAK-STAT activation, while ROS and prostaglandins amplify tissue damage. Growth factors promote synovial hyperplasia and angiogenesis, working synergistically with autoantibody–complement cascades to sustain inflammation. MMPs execute cartilage degradation, with their production regulated by multiple intracellular signaling pathways. These interconnected processes create a self-perpetuating inflammatory milieu that progressively destroys joint tissues.

#### 2.3.1. Pro-Inflammatory Factors

The upregulation of inflammatory cytokines is primarily driven by the activation of immune cells and FLSs within the synovial tissue [69]. These cells are persistently stimulated by autoantigens and immune complexes in the inflammatory environment and produce excessive pro-inflammatory cytokines such as TNF-α, IL-1β, IL-6, and IL-17 [69]. These cytokines activate downstream signaling pathways (e.g., NF-κB and JAK-STAT), thereby driving synovial hyperplasia and immune cell infiltration [8]. Additionally, the differentiation and function of Th17 cells are enhanced under the regulation of IL-23 and IL-1β [70].

The upregulation of chemokines results from sustained activation of immune cells and FLSs in the inflamed synovium [53]. Pro-inflammatory mediators stimulate these cells to produce elevated levels of chemokines, including CXCL8 (IL-8) and CCL2 (MCP-1) [53,71]. These chemokines bind to their receptors on immune cells, recruiting neutrophils, monocytes, and T cells into the synovium [60,71]. Moreover, the synovial fluid accumulation of CXCL10 correlates with its functional effects on FLSs, including enhanced invasive potential and upregulated active MMP-1 secretion [33].

The overproduction of NO and the consequent oxidative/nitrosative stress significantly contribute to the pathogenesis of RA [72]. As a key member of reactive nitrogen species, NO is catalyzed by inducible nitric oxide synthase (iNOS) under inflammatory stimulation. The excessive expression of iNOS leads to a significant increase in NO levels, which then reacts with superoxide radicals to form reactive nitrogen species such as peroxynitrite (ONOO^−^) [73]. This process triggers oxidative stress and inflammatory responses. Furthermore, NO activates guanylate cyclase to produce cyclic guanosine monophosphate (cGMP), which activates the cGMP/PKG signaling pathway, modulating inflammatory responses and oxidative stress [74]. Studies have shown that inhibiting iNOS activity or reducing excessive NO production can effectively alleviate oxidative/nitrosative stress, downregulate the cGMP/PKG signaling pathway, and restore macrophage homeostasis [75,76]. Notably, matrix degradation products in RA, particularly fibronectin fragments, further induce iNOS expression by activating the MAPK signaling pathway [73].

Prostaglandin E2 (PGE2) serves as a key mediator in driving RA pathology through diverse mechanisms [70,77]. Macrophages and FLSs are key cellular sources of PGE2, which is generated through cyclooxygenase-2 activity and exerts its biological effects by binding to four G-protein-coupled receptors (EP1, EP2, EP3, and EP4) [70,78,79,80,81]. Activation of EP2 and EP4 receptors triggers the cAMP-PKA signaling pathway, enhancing the release of pro-inflammatory cytokines such as IL-6 and TNF-α [82,83]. Furthermore, PGE2 upregulates the expression of MMPs, promotes angiogenesis, and induces synovial hyperplasia [79]. Additionally, PGE2 mediates pain signaling by activating EP receptors on neurons, leading to joint pain in RA patients [79].

#### 2.3.2. Growth Factor

Growth factors significantly contribute to the progression of RA by modulating inflammation and synovial hyperplasia. Elevated levels of growth factors in the synovial tissue and fluid correlate closely with disease activity [84]. Factors such as TGF-β and fibroblast growth factor drive the proliferation and activation of FLSs, leading to abnormal synovial tissue growth [60,85]. VEGF promotes angiogenesis, enabling leukocyte infiltration into the synovium and exacerbating hypoxia, thereby intensifying synovial inflammation [61,86]. Additionally, platelet-derived growth factor and TGF-β increase the invasiveness of FLSs [60,61]. Growth factors also interact with pro-inflammatory cytokines to create a complex signaling network that leads to inflammation [29].

#### 2.3.3. Autoantibodies and the Complement System

Autoantibodies, including RF and ACPAs, are considered central drivers of RA [87,88]. These autoantibodies recognize and bind to self-antigens, subsequently forming immune complexes that deposit in the synovial tissue. The immune complexes activate the complement system, inducing inflammation and promoting the infiltration and activation of immune cells [89]. Additionally, autoantibodies that engage Fc receptors on immune cells stimulate the release of pro-inflammatory cytokines [80,89]. In addition to their immune-modulating effects, autoantibodies can also directly activate FLSs. 

The complement system is activated in the synovial tissue by immune complexes, primarily through the classical and alternative pathways [89,90]. This activation generates anaphylatoxins (e.g., C3a and C5a), which recruit and activate inflammatory cells, driving their infiltration into the synovium [91]. C5a, upon binding to its receptor, further stimulates these cells to release pro-inflammatory cytokines [90,91,92]. Additionally, the membrane attack complex (MAC), formed during complement activation, directly damages synovial cells and chondrocytes [91]. Importantly, complement activation synergizes with autoantibodies to induce inflammation and bone erosion [93].

#### 2.3.4. MMPs

MMPs are critical mediators of joint destruction due to their ability to degrade ECM components [31]. MMPs are highly upregulated in both synovial fluid and tissues in RA, primarily stimulated by pro-inflammatory cytokines such as IL-1β, TNF-α, and IL-17 [31]. These enzymes specifically target collagen, proteoglycans, and other structural proteins in articular cartilage and bone. Beyond degrading ECM, MMPs also promote the invasion of FLSs into cartilage and bone. Furthermore, MMPs interact with other proteolytic enzymes, such as cathepsins and hyaluronidase, resulting in tissue destruction [94,95]. The increased activity of hyaluronidase in RA leads to the degradation of hyaluronic acid (HA), which compromises the viscosity and lubricating function of synovial fluid [96,97].

#### 2.3.5. Signaling Pathways

Immune-mediated inflammation in RA is amplified through cytokine-induced signaling pathways that further stimulate pro-inflammatory cytokine production [98]. These effects are predominantly mediated through the MAPK, NF-κB, PI3K/AKT, and JAK/STAT signaling pathways. The MAPK signaling pathway is activated by cytokine receptors and regulates inflammatory responses in injured joints primarily by controlling the production of additional pro-inflammatory cytokines [98]. Elevated NF-κB levels in RA synovia drive pro-inflammatory cytokine production, which further activates NF-κB, creating a vicious cycle [99]. PI3K/AKT-mediated mTOR activation suppresses autophagy in FLSs, driving synovial hyperplasia [98]. The JAK/STAT signaling pathway is activated by IL-6 and subsequently stimulates the generation of TNF-α and IL-1β [100]. It also promotes the differentiation of Th17 cells that produce IL-17 [100].

## 3. Basic Properties of Hydrogels

Hydrogels are 3D polymeric networks composed of hydrophilic polymers that exhibit exceptional water-absorbing capacities, making them highly valuable in biomedical applications. Their physicochemical properties, including swelling behavior, mechanical strength, and degradation kinetics, are controlled by their crosslinking mechanisms and network architecture.

Physical crosslinking relies on reversible, non-covalent interactions such as hydrogen bonding, hydrophobic interactions, ionic bonding, and polymer entanglement [101]. These interactions, while providing flexibility and self-healing properties, often endow hydrogels with limited mechanical strength. Although individual hydrogen bonds exhibit relatively weak binding forces, their formation into cooperative networks can significantly enhance the overall stability of the system. This dynamic weak interaction network is capable of spontaneous reorganization and self-healing under normal conditions. However, these hydrogen bonds experience rupture under external factors (e.g., mechanical stress, temperature fluctuations, or pH variations).

Chemical crosslinking forms covalent bond networks through strategies like photoinitiated polymerization, crosslinker-mediated methods, or click chemistry [101]. Compared to physical crosslinking, chemical crosslinking exhibits higher structural stability. Covalently crosslinked networks can effectively resist mechanical deformation and swelling pressure, providing materials with superior fatigue resistance. By adjusting the crosslinking density and spatial distribution of bonding sites, different mechanical response characteristics adaptable to different tissue repair needs can be obtained [102].

## 4. Design and Optimization of Hydrogels

**Figure 2:**
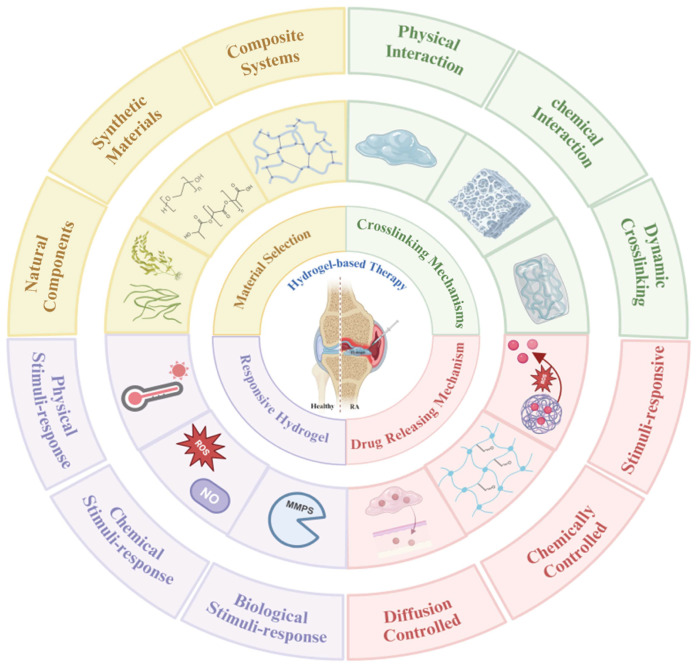
Schematic illustration of the design principles for hydrogel-based therapies, covering material selection, cross-linking methods, drug encapsulation and release, and responsive hydrogel systems. Created using https://BioRender.com.

Multifunctional systems with spatially controlled bioactivity have been constructed through the precise modulation of the material composition, crosslinking degree, and mechanical properties. These materials can modulate immune cell activity while achieving precise delivery and controlled release of therapeutic agents. The current research is focusing on developing stimuli-responsive materials, optimizing surface modification techniques, and exploring modular assembly strategies to enhance therapeutic precision and biocompatibility. This section analyzes the design principles and functional mechanisms of hydrogels, providing theoretical guidance and technical pathways for developing next-generation biomaterials (Figure 2). It should be noted that throughout this article, the term “responsive hydrogels” specifically refers to hydrogels possessing inherent responsiveness, not nanoparticles. Any discussion of responsive nanoparticles will be explicitly indicated as such.

### 4.1. Material Selection

The selection of materials is a fundamental aspect of hydrogel design, directly influencing their mechanical properties, biocompatibility, and functionality. Hydrogels can be fabricated from natural materials, synthetic materials, or composite materials. Natural materials (e.g., collagen, fibrin, and hyaluronan) are widely used in hydrogel delivery systems owing to their adjustable properties, including swelling and deswelling capacity, porosity, permeability, network structure, elasticity, and mechanical strength [102,103,104]. Given their poor mechanical properties, environmental instability, and vulnerability to microbial degradation, natural materials often require modification to enhance their mechanical strength and stability [102]. Compared to natural materials, synthetic materials [e.g., poly(ethylene glycol) (PEG) and poly(vinyl alcohol) (PVA)] have distinct advantages in forming hydrogels, including high customizability, favorable mechanical properties, and excellent water-holding capacities [102]. However, they also come with challenges like potential toxicity and biological inertness [105,106]. By combining natural and synthetic materials, composite hydrogels offer synergistic benefits while compensating for the drawbacks of the individual components [107]. Representative modifications include methacrylation of gelatin to introduce photo-crosslinkable groups for UV-triggered hydrogel formation, or functionalization of chitosan (CS) with carboxymethyl/glycol groups to optimize its solubility and gelation properties [108,109,110]. Hydrogels can be designed with customized properties through careful selection and strategic modification of these polymeric components.

### 4.2. Crosslinking-Driven Hydrogel Customization

The crosslinking degree determines the hydrogel’s properties. Higher crosslinking creates tighter networks resulting in stiffer hydrogels that swell less and degrade more slowly. Lower crosslinking forms softer hydrogels. Such hydrogels swell considerably but have relatively weak mechanical strength [102]. These structural variations give rise to two distinct pore and mesh configurations in hydrogel networks, where increased crosslinking density leads to more compact structures with reduced chain lengths [111].

To achieve precise control over these properties, contemporary hydrogel designs strategically combine three crosslinking modalities: (1) permanent covalent crosslinking; (2) dynamic covalent crosslinking (e.g., hydrazone bonds [112], disulfide bonds [113], imine bonds [114], boronate esters [115], and thioester exchange [116]); and (3) physical/supramolecular interactions (e.g., host–guest interactions using β-cyclodextrin [117] and supramolecular assemblies using ureidopyrimidinone [118]). This multimodal crosslinking approach not only enables broad stiffness tunability but also recapitulates the stress–relaxation behavior of native extracellular matrix [107,119].

### 4.3. Drug-Loading and Release Mechanisms

Drug molecules can be either directly encapsulated in the hydrogel’s porous network or physically/chemically cross-linked to the hydrogel molecular chains. Specifically, physical conjugation relies on non-covalent interactions, such as hydrogen bonding [120], electrostatic interactions [121,122], and hydrophobic effects [110]. On the other hand, chemical conjugation involves covalent bonding between drugs and the hydrogel networks through reactive groups such as esters [123], amides [124], or disulfides [125]. When these covalent bonds are subjected to hydrolysis or enzymatic cleavage, the drug encapsulated within the hydrogel matrix is gradually released [122].

The release of drugs is fundamentally governed by the complex relationship between the hydrogel’s network architecture and the physicochemical properties of the encapsulated therapeutic agents [111]. For directly encapsulated drugs, the release kinetics are primarily governed by the size relationship between drug molecules and the hydrogel’s pore architecture. While increased porosity generally enhances drug release, this effect plateaus beyond a critical porosity threshold [126]. The release behavior of drugs loaded into a hydrogel varies significantly depending on its interaction with the hydrogel network: directly encapsulated molecules diffuse freely through the porous structure, while hydrogel-conjugated drugs exhibit cleavage-dependent kinetics that respond to specific enzymatic or environmental triggers [127,128]. Furthermore, sustained drug release is linked to hydrogel degradation, where longer polymer network chains between crosslinks (higher Mc) lower the crosslinking density, thereby accelerating the degradation rate [111]. Thus, adjusting the network chain lengths can regulate the release of drugs.

Recent studies have validated the rational incorporation of nanoparticles or liposomes within hydrogels to enhance their drug-loading capacity and achieve multi-stage release patterns [121,122]. In particular, hydrogels employing responsive nanoparticles can achieve cascade-triggered intelligent drug release when responding to specific stimuli (e.g., enzymes or redox changes) [129]. Through a thorough understanding of the pathological mechanisms of diseases, hydrogels can be precisely customized. This involves the selection of drug encapsulation approaches and designs for specific stimuli-response release.

### 4.4. Responsive Hydrogels

Responsive hydrogels are designed to respond to the dynamic changes in the inflammatory microenvironment, providing targeted and on-demand therapeutic effects [16]. pH-sensitive hydrogels harness the acidic pathophysiology of inflammatory microenvironments to achieve stimulus-responsive drug release [121,130]. Thermo-responsive hydrogels experience reversible sol–gel transitions in response to temperature changes near body temperature, enabling precision thermo-activated drug localization [128]. MMP-sensitive hydrogels undergo controlled degradation in the presence of overexpressed MMPs within synovial tissues in RA [122]. NO-responsive hydrogels are tailored to release therapeutic agents in response to elevated NO levels [131]. ROS-responsive hydrogels, designed to disassemble under oxidative stress conditions, provide precision drug release in inflamed tissues [132]. By integrating these responsive mechanisms, hydrogels can achieve spatiotemporal control over drug release and maximize therapeutic efficacy [133].

## 5. Injectable Hydrogel Systems for RA Management

The current advances in RA treatment are focusing on four key approaches: (i) targeting disease mechanisms, (ii) restoring joint microenvironment homeostasis, (iii) developing smart responsive systems, and (iv) exploring innovative delivery methods. Among these, hydrogel-based IA delivery platforms have revolutionized RA management. Conventional injectable hydrogels, characterized by their sustained and controlled release of drugs, have been extensively studied for prolonging drug retention in the joint cavity and reducing systemic side effects. Many studies have indicated that conventional injectable hydrogels primarily provide drug sustained-release and physical barrier functions in RA treatment (Table 1). In contrast, novel stimuli-responsive injectable hydrogels achieve intelligent release of therapeutic agents by sensing pathological microenvironments (Table 2). Stimuli-responsive injectable hydrogels demonstrate superior therapeutic benefits through their microenvironment-responsive release mechanism, achieving on-demand drug delivery. The injectable hydrogel strategy effectively achieves an optimal balance between insufficient local drug concentration and systemic toxicity, providing new materials for efficient and safe joint-targeted therapies.

### 5.1. Immunologically Targeted Therapies for RA

The development of injectable hydrogel platforms has revolutionized RA treatment by enabling the precise modulation of pathological mechanisms. This section comprehensively reviews recent advances in hydrogel-mediated immunomodulation, with a particular focus on their regulatory effects on immune cells and cytokine networks.

#### 5.1.1. Modulation of Immune Cell Activity and Inflammation

RA is characterized by dysregulated immune cell activity. Targeting this process is a key therapeutic strategy. Hydrogels can deliver immunomodulatory agents directly to the synovium, where these agents can suppress the activation and infiltration of immune cells. For instance, a recent study demonstrated the therapeutic potential of fibroin protein hydrogels enhanced with exosomes through in situ photo-crosslinking for RA management [149]. PD-L1-expressing exosomes mediated PI3K/AKT pathway blockade, leading to attenuated Tfh cell differentiation. This attenuated Tfh response consequently impaired germinal center B cell differentiation into plasma cells.

Additional research established that mesenchymal stem cells (MSCs) function as immunomodulatory multipotent progenitors to limit RA advancement [150]. When encapsulated within ionic alginate (ALG) hydrogel networks, MSCs demonstrated enhanced viability and an enhanced immunomodulatory capacity toward DCs. The tolerogenic DCs and Treg cells were induced via the CD39/CD73/adenosine axis to alleviate inflammation.

Another study demonstrated that the dual dynamically cross-linked SPT@TPL hydrogel promoted macrophage M2 polarization for RA treatment [169]. This advanced silk fibroin/tea polyphenol (SPT) hydrogel, loaded with triptolide (TPL), employed abundant borate ester/tea polyphenol complexes that dynamically responded to and neutralized excessive ROS in arthritic joints. Its unique dual-crosslinked architecture, stabilized by hydrogen bonds and borate ester linkages, enabled controlled TPL release for over 30 days. SPT@TPL was validated to effectively attenuate oxidative stress while inducing macrophage repolarization toward the M2 anti-inflammatory phenotype. This subsequently reduced joint inflammation and facilitated cartilage regeneration.

#### 5.1.2. Cytokine Network Modulation and Inflammatory Cascade Regulation

**Figure 3:**
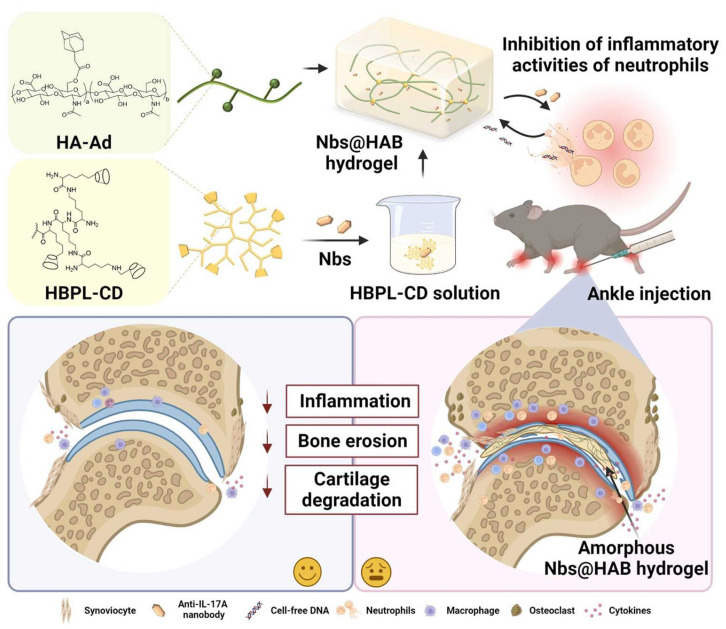
Schematic of the Nbs@HAB hydrogel for RA treatment, demonstrating dual cfDNA scavenging and IL-17A blockade to disrupt pro-inflammatory signaling cascades. Reprinted with permission from [117] (Copyright 2024, Elsevier).

The pro-inflammatory cytokines establish a pathological microenvironment in arthritic joints. Targeting the pathological crosstalk between cytokines represents a strategic approach to break the inflammatory cycle in RA. A novel platform was constructed by Wang et al. who introduced a cationic hydrogel loaded with anti-IL-17A nanobodies (Nbs) to synergistically suppress neutrophil-mediated inflammation in RA (Figure 3) [117]. The hydrogel was synthesized based on host–guest interactions, utilizing β-cyclodextrin-modified hyperbranched polylysine (HBPL-CD) and adamantane-modified hyaluronic acid (HA-Ad). Optimized for injectability and mechanical compatibility with joints, the hydrogel persistently adsorbed cell-free DNA (cfDNA) and slowly released anti-IL-17A Nbs. The released Nbs synergistically attenuated the inflammatory activities of neutrophils by inhibiting IL-17A-stimulated NETs in vivo. The hydrogel reduced the levels of pro-inflammatory cytokines and suppressed the inflammatory phenotype of neutrophils in vitro.

A similar strategy was implemented by Chen et al. who developed a thermo-responsive injectable hydrogel for pain relief and cartilage regeneration in RA [168]. This system consisted of Pluronic F127, HA, and polyglutamic acid (PGA) and was loaded with infliximab (IFX), a TNF-α inhibitor, to specifically suppress RA-associated pathological inflammation. When administered via IA injection to AIA rabbits, the IFX-loaded hydrogel showed significant therapeutic benefits. These included substantial pain relief, reduced cartilage degradation, and enhanced tissue regeneration.

A separate study found that a PC hydrogel with thermo-sensitive properties containing an HA-conjugated TLR4-blocking cyclic peptide [PC+(HA-CP)] regulated cytokine networks in RA [163]. Compared to physical mixtures [PC+(HA+CP)], this covalent system significantly enhanced peptide stability and joint retention while preserving bioactivity. Both formulations reduced inflammation and promoted cartilage repair, with the conjugated system showing superior efficacy through sustained release of the cytokine-modulating TLR4 antagonist.

The development of these immunologically targeted therapies could revolutionize RA treatment by replacing systemic immunosuppression with targeted, sustained therapeutic approaches.

### 5.2. Restoration of Joint Microenvironment Homeostasis

Emerging therapeutic strategies for RA are increasingly focusing on the dysfunctional joint microenvironment, aiming to restore physiological homeostasis. This section examines two critical approaches: (1) modulation of redox homeostasis and (2) promotion of tissue repair. Rebalancing the synovial microenvironment has two therapeutic benefits: effective inflammation control and prevention of pathological progression in RA.

#### 5.2.1. Modulation of Oxidative Stress 

**Figure 4:**
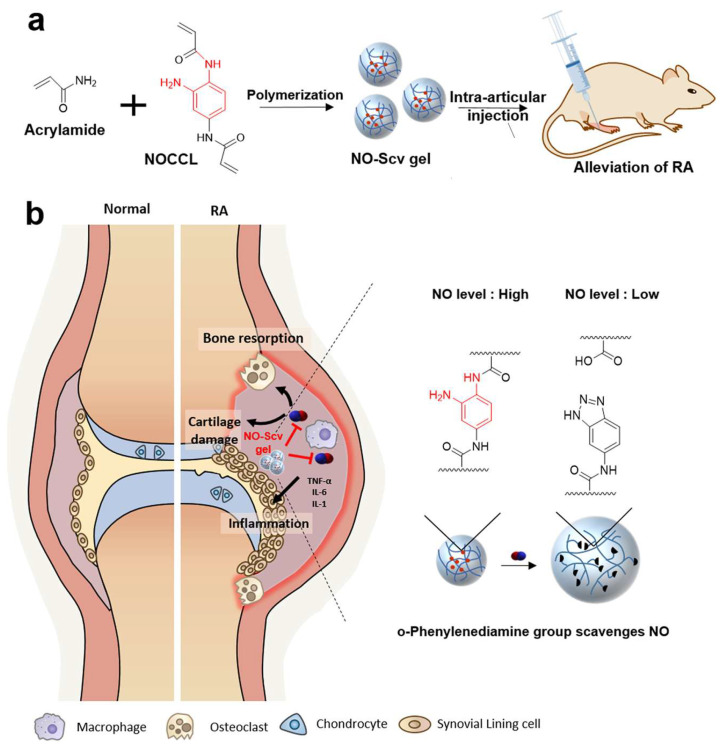
Schematic of the NO-cleavable nanogel platform enabling NO scavenging and inflammation-activated drug release for RA therapy. (**a**) Preparation and application of NO-Scv gel for treating RA. Intra-articular injection of NO-Scv gel into RA model mouse to alleviate RA by scavenging NO. (**b**) Mechanism of NO-induced inflammatory effect in RA and alleviation of RA by NO-Scv gel. NO directly damages cartilage, upregulates osteoclasts inducing deformation of bone, and further aggravates the inflammation along with proinflammatory cytokines. Reprinted with permission from [158] (Copyright 2019, American Chemical Society).

A critical aspect of joint homeostasis is the regulation of oxidative stress, which causes inflammation and tissue damage in RA. Recent advances have shown that hydrogels designed to scavenge ROS and NO exhibit remarkable therapeutic potential. Yeo et al. developed a NO-responsive and NO-scavenging nanosized hydrogel (NO-Scv gel) for the treatment of RA (Figure 4) [158]. The NO-Scv gel was prepared through solution polymerization of acrylamide and an NO-cleavable cross-linker. The NO-Scv gel demonstrated efficacy in reducing inflammation through NO scavenging while exhibiting excellent biocompatibility. 

Similarly, Xu et al. developed an advanced injectable hydrogel with dual functions of ROS scavenging and self-regulated MTX release for RA treatment [132]. Composed of HA-PEG matrices loaded with both mesoporous polydopamine nanoparticles and MTX, the hydrogel demonstrated a potent ROS elimination capacity. This system achieved prolonged, staged, and self-regulated MTX release, ensuring persistent therapeutic efficacy and responsive to disease activity. The single IA injection of the hydrogel showed potent anti-inflammatory effects in vivo, outperforming conventional treatments.

Another key strategy was developed by Wang et al. who reported a novel multifunctional bio-responsive hydrogel system based on tripolycerol monostearate. This hydrogel was designed to regulate the hypoxic joint microenvironment and deliver psoralen for RA management [156]. The hydrogel, co-loaded with psoralen and calcium peroxide, responded to arthritis-related MMPs to release drugs and oxygen (O2) in vitro, thereby addressing hypoxia and inflammation. The psoralen gel also demonstrated enhanced therapeutic effects in vivo.

#### 5.2.2. Modulation of Tissue Repair and Regeneration

**Figure 5:**
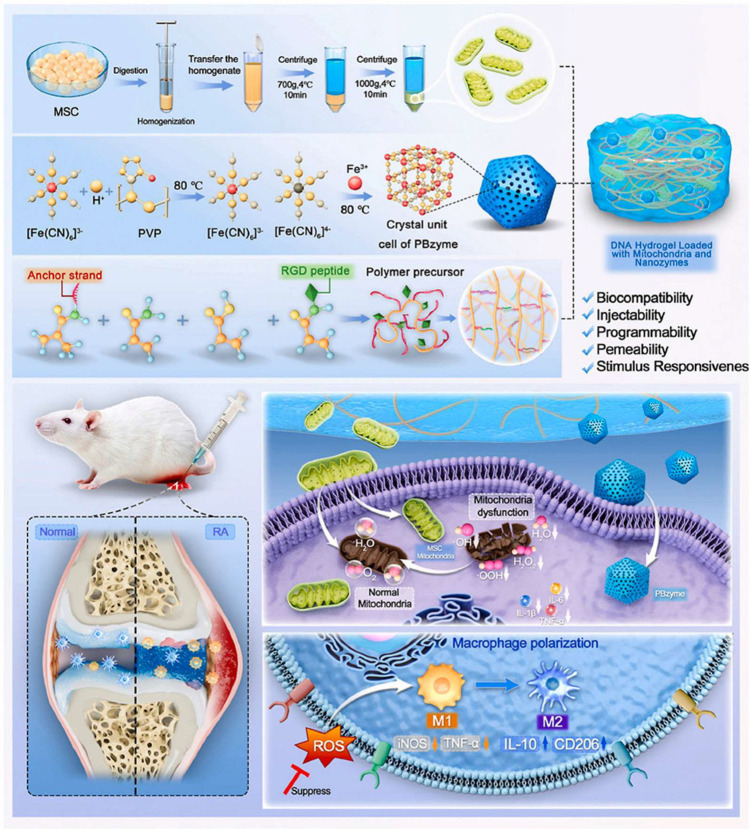
Schematic of the polymer-modified DNA hydrogel co-loaded with Prussian blue nanozymes and functional mitochondria, illustrating the combined anti-inflammatory and pro-regenerative actions that enhance chondrocyte function for joint repair. Reprinted under the terms of the CC BY-NC-ND 4.0 license from [151] (Copyright 2025, the authors, published by Elsevier).

Hydrogels can exert therapeutic effects in RA through tissue repair and regeneration mechanisms. By promoting these regenerative processes, they simultaneously suppress pathological inflammation and angiogenesis. Through their ability to mimic the ECM, hydrogels provide a scaffold that facilitates cell adhesion, proliferation, and differentiation. For example, in the work by Wang et al., a polymer-modified DNA hydrogel co-loaded with Prussian blue nanozymes and functional mitochondria was developed to promote chondrocyte proliferation and differentiation (Figure 5) [151]. The combination of nanozyme catalytic activity with the mitochondrial therapy’s regenerative capabilities endowed the hydrogel with dual therapeutic effects, reducing inflammation while enhancing tissue repair.

Similarly, the work by Bahatibieke et al. showed that a polyurethane–gelatin composite hydrogel scaffold (TP@GSPU) concurrently attenuated inflammatory responses and promoted cartilage regeneration in RA [170]. Through its unique sea–island micelle structure, TP@GSPU achieved efficient loading and controlled release of triptolide (TP). This hydrogel regulated macrophage-mediated inflammatory responses to improve the joint microenvironment. Simultaneously, its gelatin component provided structural support for cartilage regeneration. TP@GSPU demonstrated dual therapeutic efficacy in vivo by mitigating synovial inflammation and promoting cartilage repair.

Through modulating the inflammatory cascade and promoting tissue regeneration, the injectable hydrogel delivery system effectively enhanced therapeutic safety and efficacy.

### 5.3. Smart Hydrogels with On-Demand Microenvironment Responsiveness

Smart hydrogels can be designed to release therapeutic agents in response to specific pathological stimuli (e.g., acidic pH, elevated ROS levels, or overexpressed enzymes) within inflamed joints. This section highlights two innovative strategies: (i) pathological factor-responsive systems and (ii) prodrug-loaded nanocomposite hydrogels, which combine nanoparticle technology with stimuli-responsive release mechanisms and have demonstrated on-demand therapeutic potential for RA management.

#### 5.3.1. Pathological Factor-Triggered Hydrogel Platforms

**Figure 6:**
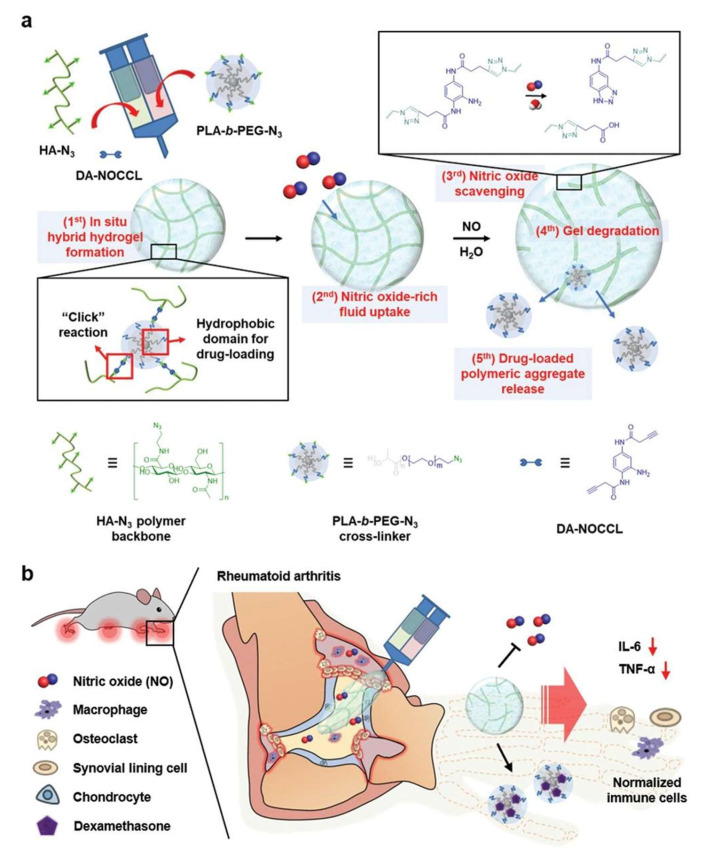
Schematic showing the M-NO gel’s microenvironment-responsive behavior through NO-triggered DA-NOCCL crosslinking, which mediates both NO scavenging and dexamethasone release for enhanced RA treatment. (**a**) Schematic illustration and chemical structure of the polymeric aggregate-embodied hybrid nitric oxide (NO)-scavenging “click” hydrogel (M-NO gel). Self-healable in situ hydrogel cross-linked with DA-NOCCL (N,N-(2-amino-1,4-phenylene) dipentyn-4-amide) reacts with and scavenges NO to further release drug-loaded polymeric aggregates for the combinatorial treatments. (**b**) Combinatorial treatment of rheumatoid arthritis (RA) with M-NO gel. M-NO gel directly scavenges overproduced NO and releases anti-inflammatory dexamethasone, depending on the severity of the diseases with NO concentration as a hallmark. Reprinted with permission from [157] (Copyright 2021, Wiley).

Targeting the pathological microenvironment of RA, researchers have developed stimuli-responsive hydrogel platforms that exploit disease-specific factors (e.g., increased ROS and NO levels, overexpressed enzymes, acidic conditions, and elevated temperatures) to achieve precision therapy. These responsive hydrogels enable on-demand drug release, prolonging therapeutic retention in inflamed joints. For example, inflammation-responsive hydrogels, such as OD-PP@SeNPs, have been developed to scavenge ROS and regulate pH, promoting macrophage polarization toward the anti-inflammatory M2 phenotype [127]. In a similar vein, an injectable and NO-responsive polymeric aggregate-embodied hybrid hydrogel (M-NO gel) was designed to co-deliver dexamethasone for the combinatorial treatment of RA (Figure 6) [157]. Along the same lines, a recent study developed an enzyme-responsive hydrogel platform to deliver triamcinolone acetonide for the treatment of inflammatory arthritis, enabling on-demand drug release during arthritis flares [155].

Several smart delivery systems also demonstrate specific responsiveness to the acidic pH and increased temperature in RA. As a representative example, Liu et al. designed a pH-responsive hydrogel for the treatment of RA [159]. The therapeutic platform employed hollow honeycomb electroacupuncture needles (HC-EA) loaded with a melittin hydrogel (MLT-Gel). The hydrogel, composed of CS, sodium β-GP, and HA, was engineered to respond to the acidic RA microenvironment, enabling drug release at the lesion site. The hollow honeycomb structure of the acupuncture needles provides a high drug-loading capacity, integrating acupuncture stimulation and melittin therapy. The MLT-Gel@HC-EA treatment restored the balance of Th17/Treg-mediated immunity and reduced the release of pro-inflammatory cytokines (TNF-α, IL-6, and IL-1β), thereby alleviating inflammation at the lesion site. 

Temperature-responsive hydrogel systems represent another important class of smart hydrogel platforms. Yin et al. developed a thermosensitive hydrogel (D-NGel) co-loaded with indomethacin and MTX nanoparticles for synergistic RA treatment [171]. The hydrogel, composed of 27% F127 and 10% F68, exhibited rapid sol–gel transition at 33 °C within 15 s. This hydrogel sustained drug release for 72 h while maintaining good biocompatibility. The formulation significantly alleviated joint inflammation and bone erosion, as evidenced by reduced swelling and suppressed inflammatory cytokine levels in the synovial fluid. 

These systems provide rapid coverage of inflamed joint surfaces while establishing a robust platform through on-demand drug release and sustained therapeutic retention, enabling both immediate symptom relief and long-term disease control.

#### 5.3.2. Prodrug-Mimicking Nanoparticle-Loaded Hydrogel Platforms

This integrated platform combines stimuli-responsive prodrug nanoparticles with hydrogel carriers for controlled drug delivery. The nanoparticles respond to pathological microenvironments, while the hydrogel acts as a protective reservoir that prevents premature clearance and sustains release at the target site. This system exhibits a sequential release mechanism: progressive hydrogel degradation mediates nanoparticle delivery, followed by stimulus-triggered drug release from the accumulated nanoparticles.

Du et al. presented an innovative phospholipid-mimic artemisinin prodrug (ARP) incorporated within a liposome-embedded hydrogel nano/microsphere for RA therapy [172]. The injectable formulation combines ARP with MTX in “drug-in-drug” liposomes stabilized by a hydrogel matrix, enabling sustained IA release. Following local administration, the released liposomes specifically target synovial macrophages and fibroblasts. The phospholipid-mimic ARP is stable extracellularly but undergoes rapid lysosomal conversion to active dihydroartemisinin post-internalization, achieving precise in situ activation.

Yang et al. introduced an innovative hierarchical drug delivery system for RA treatment, combining hyaluronic acid (HA) microparticles with MTX-conjugated peptide dendrimer nanoparticles [173]. The injectable HA-based system exhibited pH-responsive release in the acidic synovial environment. The conjugated nanoparticles enhanced intra-cellular drug delivery and promoted macrophage polarization from the pro-inflammatory M1 to the anti-inflammatory M2 phenotype. In vivo evaluations revealed that this combinatorial strategy significantly reduced joint inflammation, preserved cartilage integrity, and improved bone mineral density.

An advanced IA delivery system was created by Abou-ElNour et al. through the strategic integration of two functional components: thermo-responsive star-shaped poly copolymers and triamcinolone acetonide-bearing PLA/mPEG-PDL microparticles for optimized RA therapy [174]. Two copolymer formulations demonstrated concentration-dependent gelation at physiologically relevant temperatures. The composite system exhibited prolonged anti-inflammatory effects, which were attributed to the combined advantages of thermo-responsive hydrogel retention and microparticle-mediated controlled drug release.

Combining multiple therapeutic agents in hydrogels can further enhance their efficacy. Du et al. developed a nanocomposite hydrogel system using CS and β-GP to encapsulate acid-responsive prodrugs of lenalidomide and hesperidin for the localized treatment of RA [129]. The hydrogel system demonstrated sustained pH-responsive drug release and effectively alleviated synovial inflammation and joint destruction in vivo through targeting PI3K/AKT signaling to reduce pro-inflammatory cytokine secretion and inhibit angiogenesis. 

### 5.4. Innovative Hydrogel Therapies: Gas-, Sound-, Light-, and 3D Printing-Based Approaches

Beyond traditional hydrogel-based treatments, emerging strategies incorporating gas therapy, sonodynamic therapy (SDT), and phototherapy/photodynamic therapy (PTT/PDT) have demonstrated significant potential in addressing RA. Compared with traditional therapies, multimodal hydrogel systems incorporating novel strategies have demonstrated enhanced efficacy. Nevertheless, these emerging technologies present several challenges, including precise control of gas release, potential tissue damage from excessive ROS generation during SDT/PDT, and the limited penetration depth of light-based therapies in joints.

#### 5.4.1. PTT/PDT

Light-responsive hydrogels provide a controllable and precise therapeutic platform. Near-infrared (NIR) light can trigger hydrogel degradation or activate embedded nanoparticles to release drugs or generate ROS to eliminate FLSs to suppress inflammation. A related advancement by Chiang et al. involved the design of a photothermal-responsive hydrogel based on methylcellulose for injectable RA treatment [153]. The hydrogel incorporated strontium ranelate and sodium chloride. The photothermal properties of polypyrrole–polyethylenimine nanoparticles promote blood circulation and suppress inflammation. The hydrogel exhibited enhanced mechanical strength and sustained release of SrR. The photothermal effect induced by NIR irradiation increased local blood flow while alleviating inflammation and promoting cartilage repair. This system combines photothermal therapy with controlled drug release to improve RA treatment and reduce the frequency of injections.

Similarly, Rui et al. developed a prolonged O_2_/Ca^2+^-supporting PTT hydrogel for RA treatment [167]. The hydrogel contained calcium peroxide (CaO_2_), which served as both an O_2_ and calcium ion (Ca^2+^) source. This dual functionality supported PDT through a continuous O2 supply for sustained singlet O_2_ (^1^O_2_) production. The generated ^1^O_2_ effectively eliminated FLSs while inducing immunogenic cell death. Moreover, the released Ca^2+^ triggered mitochondrial Ca^2+^ overload and endoplasmic reticulum Ca^2+^ disorder, thereby promoting apoptosis and cartilage regeneration. This multifunctional hydrogel suppressed abnormal FLS proliferation and mitigated local hypoxia.

**Figure 7:**
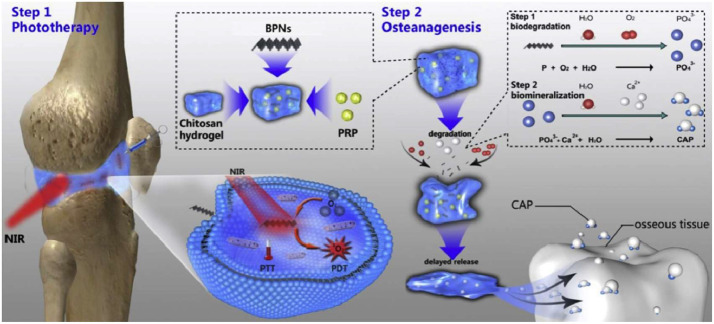
Schematic of the multifunctional thermo-responsive hydrogel system based on black phosphorus nanosheets (BPNs), demonstrating NIR-triggered phototherapy for FLS elimination while promoting tissue regeneration through PRP-derived growth factors and CS-mediated cartilage protection. Reprinted with permission from [162] (Copyright 2020, Elsevier).

Another example is the work by Pan et al. who developed a novel multifunctional thermo-responsive hydrogel system based on black phosphorus nanosheets (BPNs) for RA treatment (Figure 7) [162]. The hydrogel, comprising BPNs, CS, and platelet-rich plasma (PRP), utilized the phototherapeutic properties of BPNs to eliminate FLSs under NIR irradiation. PRP contributed bioactive factors that enhanced tissue regeneration through growth factor signaling, while CS provided viscoelastic properties for articular cartilage protection. Furthermore, the system’s calcium-free phosphorus component stimulated biomineralization and promoted osteanagenesis. The in vitro and in vivo experiments revealed the multifunctional capacity of the thermosensitive BPNs/CS/PRP hydrogel, which simultaneously promoted biomineralization, facilitated MSC adhesion, and enabled ROS delivery to pathogenic FLSs.

Wu et al. employed an alternative strategy by introducing an injectable and pH-sensitive peptide hydrogel to co-deliver MTX and bismuthene nanosheets/polyethyleneimine (BiNS/PEI) for RA treatment [121]. This platform integrates MTX’s anti-inflammatory and anti-apoptotic properties with BiNS/PEI’s ability to eliminate FLSs through combined PDT and PTT. The system demonstrated synergistic anti-inflammatory and FLS-eliminating effects, achieving robust therapeutic outcomes in vitro and in vivo. Expanding on this work, they subsequently designed another hydrogel system incorporating siRNA/MTX-polyethyleneimine and bismuthene nanosheets/MTX–polyethyleneimine [130]. This enhanced platform employed RNA interference (RNAi) to inhibit key immune pathways (e.g., NF-κB and MAPK-p38). Through the synergistic application of PDT and PTT, the system successfully targeted and eliminated hyperactive FLSs. The combined therapeutic strategy synergistically suppressed inflammation and selectively eliminated FLSs.

#### 5.4.2. Gas Therapy

Hydrogel-based gas therapy represents a novel direction for RA. Hydrogels that release therapeutic gas [such as hydrogen (H_2_), hydrogen sulfide (H_2_S), or O_2_] can reduce inflammation, scavenge ROS, and promote tissue repair [140]. As a representative example, Geng et al. developed a self-healing injectable hydrogel that releases H_2_S for RA treatment [141]. The hydrogel effectively eliminated excess NO and delivered anti-inflammatory H_2_S, creating a favorable environment for bone regeneration. This hydrogel demonstrated efficient drug loading and controlled release, achieving enhanced therapeutic outcomes through synergistic effects.

Xu et al. designed a self-propelled magnesium–hyaluronic acid micromotor for RA treatment [175]. This system continuously releases H_2_ gas to attenuate oxidative stress and downregulate inflammatory factors. The micromotors were fabricated by asymmetrically coating magnesium (Mg) microparticles with an HA-loaded hydrogel and a poly(lactic-co-glycolic acid) layer, continuously generating H_2_. This design significantly enhanced the micromotors’ ability to scavenge ROS and reduce pro-inflammatory cytokine levels. The micromotors achieved precise positioning within the joints through ultrasound-guided real-time visualization of H2 bubbles in vivo.

Zhao et al. developed a nanozyme-reinforced hydrogel for RA treatment using gas therapy to restore joint microenvironment and enhance prosthetic interface osseointegration [140]. The hydrogel scavenged over-produced ROS and synergistically generated dissolved O_2_, addressing the challenges of hypoxia and oxidative stress. This injectable hydrogel served as a delivery vehicle for bone marrow-derived mesenchymal stem cells (BMSCs), protecting the cells from ROS-mediated damage and hypoxia-induced osteogenic limitations. By encapsulating BMSCs, the hydrogel achieved two functions: it could suppress local inflammatory cytokines and improve osseointegration.

Gas therapy offers distinct therapeutic advantages for RA treatment. The low molecular weight and high diffusibility of therapeutic gases enable deep penetration into inflamed synovial tissues. These gases have multiple functions: mitigating oxidative stress, regulating inflammation, and enhancing tissue regeneration.

#### 5.4.3. SDT

**Figure 8:**
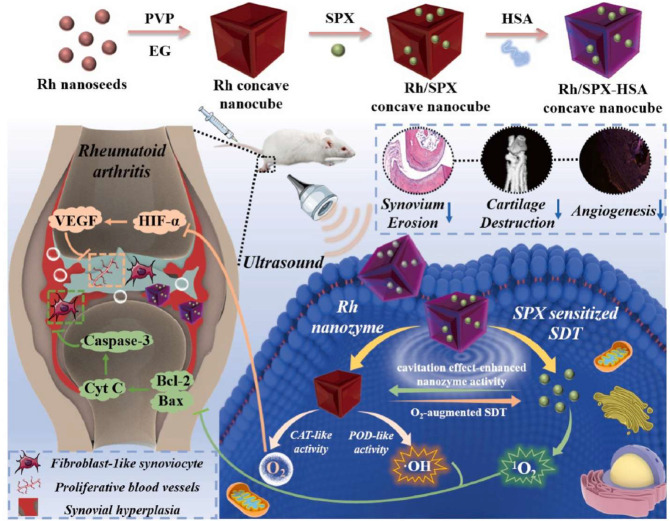
Schematic of the concave-cubic rhodium nanozyme loaded with sparfloxacin and human serum albumin, illustrating ultrasound-triggered ROS generation and catalytic hypoxia alleviation for enhanced SDT in RA. Reprinted with permission from [177] (Copyright 2021, Elsevier).

Combining hydrogels with ultrasound-responsive agents holds significant potential for RA treatment. Hydrogel-based SDT has not yet been extensively explored, but the idea is theoretically sound. Hydrogels could serve as an ideal platform for localized and sustained SDT. Ultrasound-triggered hydrogels can release drugs or generate ROS to eliminate overactive FLSs and reduce inflammation. Moreover, significant advancements have been made in related nanodelivery systems, which provide valuable insights for future hydrogel-based SDT applications (Figure 8) [176,177]. For example, Li et al. proposed a novel approach to enhance SDT for RA by addressing the challenges of sonosensitizer accumulation and synovial hypoxia [177]. They constructed a concave-cubic rhodium (Rh) nanozyme loaded with the sonosensitizer sparfloxacin (SPX) and human serum albumin. Under ultrasound activation, SPX induced mitochondrial dysfunction and excessive ROS production, effectively suppressing FLSs. Simultaneously, the Rh nanozyme exhibited peroxidase (POD)- and CAT-like enzyme activities, alleviating hypoxia in the synovial microenvironment and enhancing SDT efficacy by increasing ^1^O_2_ levels. The ultrasonic cavitation effect further amplified the nanozyme’s activity, creating a mutually reinforced SDT mechanism. 

Similarly, Wu et al. explored the potential of SDT in RA by leveraging nanoengineered macrophages as a targeted delivery system [176]. They constructed macrophage-mediated carriers by loading protoporphyrin (PPIX), a sonosensitizer, onto Fe_3_O_4_ nanoparticles, which were then internalized by macrophages. Upon intravenous injection, these carriers migrated to inflamed RA tissues, guided by cytokine signals. This system generated ROS under ultrasound activation, leading to macrophage damage and the subsequent release of Fe_3_O_4_-PPIX. This process induced ferroptosis through both the synergistic effects of Fe3O4 and extensive damage to hyperplastic FLSs and infiltrated inflammatory cells. 

While hydrogel-based SDT is still in its early stages, these advancements in nanodelivery systems provide valuable insights and pave the way for future hydrogel applications in SDT for RA.

#### 5.4.4. Printing-Based Therapy

**Figure 9:**
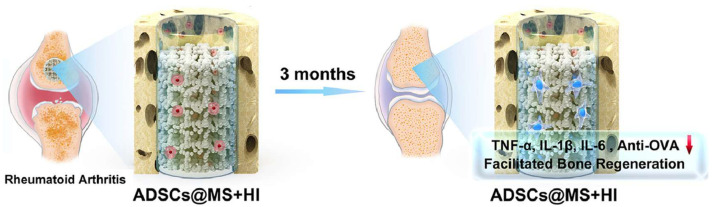
Schematic of the hybrid system combining 3D-printed porous metal scaffolds with infliximab-based hydrogels for RA treatment, demonstrating integrated mechanical support and creating a regenerative microenvironment for combined bone repair and anti-inflammatory therapy. Reprinted with permission from [152] (Copyright 2021, Elsevier).

Three-dimensional printing technology has emerged as a transformative tool in the development of hydrogel-based therapies for RA. By leveraging 3D printing, researchers can fabricate hydrogel scaffolds with tailored architectures that mimic the native joint microenvironment, enabling localized and sustained drug delivery. For example, Zhao et al. developed an innovative hybrid system for RA treatment by combining 3D-printed porous metal scaffolds with infliximab-based hydrogels, enabling simultaneous bone defect repair and stem cell-based therapeutic intervention (Figure 9) [152]. The system provided strong mechanical support, while the hydrogels formed a beneficial microenvironment for ADSCs. This combined approach significantly attenuated inflammation while enhancing cartilage regeneration and bone repair.

Elshabrawy et al. developed a triple-layered platform for RA management using 3D printing, electrospinning, and electrospraying [178]. The platform integrated three functional layers: (i) an electrospun PCL nanofiber layer for mechanical support; (ii) a composite layer of diclofenac-loaded PVA nanoparticles and nanofibers; and (iii) a 3D-printed hydrogel mesh layer with rosuvastatin-loaded lipid nanocapsules. These components created an integrated system that effectively suppressed white blood cell activity, reduced cartilage degradation, and protected synovial tissues from mechanical damage.

Currently, 3D printing technology has made it possible to fabricate hydrogel scaffolds that can highly resemble the natural joint microenvironment. These scaffolds offer two primary therapeutic advantages: customized mechanical reinforcement and controlled anti-inflammatory drug release. Through sustained medication delivery and structural stability at injury sites, they can establish an optimal environment for gradual tissue repair. These advantages bring distinctive options for RA management.

## 6. Challenges and Insights into Future Developments

Injectable hydrogels show significant promise for RA management, yet face considerable challenges in both laboratory development and clinical translation. From a materials perspective, the primary challenge lies in balancing mechanical strength with degradation kinetics—rapid degradation risks uncontrolled drug release while slow degradation may trigger immunological rejection. Although inherently biocompatible, hydrogel byproducts can potentially induce immune reactions or chronic inflammation. Researchers must achieve sustained (weeks-to-months), targeted drug delivery to synovial cells within dynamic inflammatory environments, a process complicated by pathological joint changes like synovial hyperplasia that impair hydrogel distribution and lesion coverage. The literature documents numerous successful implementations of such sustained-release systems, with certain hydrogel formulations demonstrating articular retention periods extending beyond one month [128,169]. These retention characteristics are achieved through careful optimization of hydrogel composition parameters such as polymer molecular weight and crosslinking density, as well as the incorporation of microenvironment-responsive elements that enable inflammation-triggered degradation. Beyond these immediate challenges, the long-term IA effects of hydrogel therapies on tissue remodeling and functional recovery remain critical considerations, as prolonged material presence may influence synovial regeneration, cartilage homeostasis, and joint biomechanics in incompletely understood ways. For instance, while certain hydrogel formulations demonstrate chondroprotective effects through controlled anti-inflammatory factor delivery, extended material residence could potentially impede natural synovial regeneration or disrupt cartilage homeostasis. Crucially, effective inflammation suppression does not consistently correlate with functional joint restoration. The translation of short-term anti-inflammatory effects into sustained functional improvements requires further investigation using advanced preclinical models that evaluate both structural and functional outcomes over clinically relevant periods.

The translational pathway presents additional hurdles: undefined cGMP standards for responsive hydrogels, technical–economic challenges in scaling complex formulations (e.g., gene-editing hydrogels), and a lack of regulatory frameworks for evaluating smart microenvironment-responsive systems. Critical knowledge gaps remain in long-term (≥12 month) biocompatibility assessment and standardized preclinical models. These challenges demand intensified collaboration between academia, industry, and regulators to establish clear development pathways.

The future development of hydrogels should focus on multifunctional systems integrating nanomaterials, bioactive molecules, or gene-editing tools, although their complexity raises scale-up and regulatory challenges. Smart hydrogels with stimuli-responsive components enable precise drug release but require rigorous quality control, while combination approaches with cell/gene therapies demand novel regulatory frameworks. Emerging formulations like peptide gels and 3D-printed implants are expanding therapeutic applications despite the translational hurdles [179].

Currently, few hydrogel formulations specifically target rheumatoid arthritis (RA), unlike osteoarthritis, largely due to RA’s systemic nature requiring broader immune modulation. An exception is repository corticotropin injection (Acthar^®^ Gel), which was approved as adjunctive therapy for administration in patients with persistent RA disease activity. It has been evaluated in several clinical trials for RA (NCT02919761, NCT03511625, NCT03082573, NCT02434757, and NCT01966718) [180].

Although RA is a systemic disease, localized hydrogel therapies offer distinct advantages such as targeted delivery, reduced systemic toxicity, and potential for combination with immunomodulators. Nevertheless, formulation standardization remains challenging. These innovations hold promise for bridging the gap between localized delivery and systemic therapeutic needs in RA management, complementing existing biologics and small-molecule therapies, and thereby further expanding the therapeutic potential of hydrogels.

## Figures and Tables

**Table 1:** Properties and applications of conventional hydrogels in RA.

Hydrogel Material(s)	Hydrogel Formation Mechanism	Bioactive Agent(s)	Inflammatory Arthritis Model(s)	Reference
Collagen and HA	Electrostatic interactions	BP-HA/Zn-nrBCP	CIA rabbit model; RA femur defect model	[134]
DNase-OHA/CMCS	Schiff base reaction	MTX/DNase I	CIA mouse model	[135]
Gelatin/PEGDA	Phase separation	Fol-PDA@Leonurine NPs	CIA rat model	[136]
Click-HA	Click chemistry	TLR2-antag-Pep2	CIA rat model	[137]
mPEG-(PCL-ran-PLLA)-COOH	Electrostatic interactions	MINO/SSZ	CIA rat model	[138]
HAMA	UV-initiated crosslinking	Gene/ART-NMPs	modified AIA rat model	[139]
CS/β-GP	Electrostatic/H-bond interactions	DCF-Alg-MS	AIA rabbit model	[120]
HA-Hyd/HA-Ald	Schiff base reaction	MnCoO nanozyme	OVA/Freund’s adjuvant-induced RA rabbit model	[140]
HA-SH/DNRS copolymer	Click chemistry	MTX/H_2_S	CIA rat model	[141]
β-CD-HP/Ada-HA	Host–guest interactions	Anti-IL17A-Nb	CIA mouse model	[117]
Dendritic PLL/HA	Schiff base reaction	ADSCs	CIA mouse model	[142]
Nap-DFDFDEGPIRRSDS	Non-covalent interactions	Met@hCuS-NPs	AIA rat model	[143]
β-CD-GO-HA/Ada-4armPEG	Non-covalent interactions	Chicoric acid	AIA rat model	[144]
Dex/DCF/CaCl_2_	Ionic coordination	DMT/DCF	AIA rat model	[145]
HA-Tyr	Radical crosslinking	DMT	CIA rat model	[146]
HA-AAc/(SH-PEG-SH)	Michael addition	Iguratimod	CIA mouse model	[147]
Silk fibroin	Photo-crosslinking	OE-MSC-Exos	CIA rat model	[148]
Alginate/CaCl_2_	Ionic crosslinking	BMSCs	CIA mouse model	[149]
CS/β-GP	H-bonding/electrostatics	LEN/HSP	CIA mouse model	[150]
DNA/PAAm matrix	Watson–Crick base pairing	PBzyme/Mito	CIA rat model	[129]
HA-Hyd/HA-Ald	Schiff base reaction	Infliximab	CIA mouse model	[151]
Methylcellulose	Hydrophobic interactions	Sr-ranelate	AIA rabbit model	[152]
Collagen and HA	Electrostatic interactions	BP-HA/Zn-nrBCP	Zymosan-induced RA rat model	[153]

Abbreviations: HA (hyaluronic acid), OHA (oxidized HA), AIA (adjuvant-induced arthritis), CIA (collagen-induced arthritis), RA (rheumatoid arthritis), OVA (ovalbumin), CMCS (carboxymethyl chitosan), PEGDA (polyethylene glycol diacrylate), HAMA (HA methacrylate), CS (chitosan), β-GP (β-glycerophosphate), PLL (polylysine), Dex (dextran), DCF (diclofenac sodium), PAAm (polyacrylamide), GO (graphene oxide), 4armPEG (4-arm polyethylene glycol), BP-HA (bisphosphonate-HA), Zn-nrBCP (zinc-doped nanorod biphasic calcium phosphate), MTX (methotrexate), DNase I (deoxyribonuclease I), Fol-PDA (folate–polydopamine), NPs (nanoparticles), TLR2-antag-Pep2 (Toll-like receptor 2-antagonizing peptide 2), MINO (minocycline hydrochloride), SSZ (sulfasalazine), ART (artemisinin), NMPs (nano-microplexes), Alg-MS (alginate microspheres), H_2_S (hydrogen sulfide), anti-IL17A-Nb (anti-interleukin-17A nanobody), ADSCs (adipose-derived stem cells), Met (metformin hydrochloride), hCuS (hollow copper sulfide), DMT (dexamethasone), OE-MSC (olfactory ecto-mesenchymal stem cells), Exos (exosomes), BMSCs (bone marrow mesenchymal stem cells), LEN (lenalidomide), HSP (hesperidin), PBzyme (Prussian blue nanozymes), Mito (mitochondria), s.c. (subcutaneous).

**Table 2:** Design strategies and applications of stimuli-responsive hydrogels in RA.

Hydrogel Material(s)	Hydrogel Formation Mechanism	Internal Stimulus	Bioactive Agent(s)	Inflammatory Arthritis Model	Reference
9AA-SA-AA	Hydrophobic interactions	MMPs	SA-AA/9AA amphiphiles	CIA rat model	[154]
TGMS	Hydrophobic interactions	MMPs	Triamcinolone acetonide	K/BxN mouse model	[155]
Gelatin/Soy lecithin	Chemical crosslinking	MMPs	MTX	AIA rat model	[122]
TGMS	Hydrophobic interactions	MMPs	Psoralen/CaO_2_	CIA rat model	[156]
PEG-PLA-N_3_/HA-N_3_	Click chemistry	NO	DMT	CIA mouse model	[157]
AAm/NO-cleavable linker	Polymerization	NO	AAm/linker	CIA mouse model	[158]
DHP/AAm	Radical polymerization	NO	MTX	AIA rat model	[131]
IOK peptide	Physical interactions	pH	MTX/Bi nanosheets	AIA rat model	[121]
CS/β-GP/HA	H-bonding/hydrophobic	pH	Melittin	CIA mouse model	[159]
Nap-FFKRGH	π-π stacking	pH	siRNA/MTX-PEI/bismuthene	CIA rat model	[130]
HA/PEG	Bioorthogonal chemistry	ROS	MPDA/MTX	CIA rat model	[132]
DA-HA/SCS	Schiff base	ROS/pH	MTX	CIA rat model	[160]
PBA-PLL/Odex/SeNPs	Multiple bonding interactions	ROS/pH	SeNPs	CIA rat model	[127]
CS/Gly/borax	Intermolecular interactions	Temperature	DMT	CIA rat model	[161]
PRP-CS	Electrostatic/H-bonding	Temperature	BP nanosheets	CIA mouse model	[162]
HPMAm	Thermal phase transition	Temperature	DMT	AIA rat model	[128]
mPEG-PCL-PLLA	Hydrophobic interactions	Temperature	Phage peptides	CIA rat model	[163]
CS/β-GP/OCS	Schiff base	Temperature	Apoferritin/siHMGB1	CIA rat model	[164]
CS/β-GP	Non-covalent interactions	Temperature	Triptolide	CIA mouse model	[110]
PF127	Thermogelation	Temperature	ICG/CAT/SIN	CIA mouse model	[165]
Collagen/poloxamer	Sol–gel transition	Temperature	Melatonin	AIA rat model	[166]
CS/β-GP	Non-covalent interactions	Temperature	Crocin I/Dex-liposomes	AIA rat model	[160]
PLGA-PEG-PLGA	Thermogelation	Temperature	CaO_2_	CIA rat model	[167]
PF127/HA/PGA	Hydrophobic interactions	Temperature	Infliximab	AIA rabbit model	[168]

Abbreviations: 9AA-SA-AA (9-aminoacridine-6-O-stearoyl-L-ascorbic acid), TGMS (triglycerol monostearate), MTX (methotrexate), DMT (dexamethasone), MMPs (matrix metalloproteinases), NO (nitric oxide), ROS (reactive oxygen species), CIA (collagen-induced arthritis), AIA (adjuvant-induced arthritis), K/BxN (KRN serum transfer), HA (hyaluronic acid), CS (chitosan), β-GP (β-glycerophosphate), PEG (polyethylene glycol), PLA (polylactic acid), AAm (acrylamide), DHP (dihydropyridine), PEI (polyethylenimine), MPDA (mesoporous polydopamine), SCS (succinate-modified chitosan), PBA (phenylboronic acid), PLL (polylysine), Odex (oxidized dextran), SeNPs (selenium nanoparticles), PRP (platelet-rich plasma), BP (black phosphorus), HPMAm (N-(2-hydroxypropyl)methacrylamide), OCS (oxidized chondroitin sulfate), ICG (indocyanine green), CAT (catalase), SIN (sinomenine), PGA (polyglutamic acid).

## Data Availability

Not applicable.

## References

[1] Di Matteo A., Bathon J.M., Emery P. (2023). Rheumatoid arthritis. Lancet.

[2] Gravallese Ellen M., Firestein Gary S. (2023). Rheumatoid Arthritis—Common Origins, Divergent Mechanisms. N. Engl. J. Med..

[3] Finckh A., Gilbert B., Hodkinson B., Bae S.-C., Thomas R., Deane K.D., Alpizar-Rodriguez D., Lauper K. (2022). Global epidemiology of rheumatoid arthritis. Nat. Rev. Rheumatol..

[4] James S.L., Abate D., Abate K.H., Abay S.M., Abbafati C., Abbasi N., Abbastabar H., Abd-Allah F., Abdela J., Abdelalim A. (2018). Global, regional, and national incidence, prevalence, and years lived with disability for 354 diseases and injuries for 195 countries and territories, 1990–2017: A systematic analysis for the Global Burden of Disease Study 2017. Lancet.

[5] Negi S., Tandel N., Sharma P., Kumar R., Tyagi R.K. (2023). Aceclofenac and methotrexate combination therapy could influence Th1/Th17 axis to modulate rheumatoid-arthritis-induced inflammation. Drug Discov. Today.

[6] Chapa-Villarreal F.A., Stephens M., Pavlicin R., Beussman M., Peppas N.A. (2024). Therapeutic delivery systems for rheumatoid arthritis based on hydrogel carriers. Adv. Drug Deliv. Rev..

[7] Buckner J.H. (2025). Antigen-specific immunotherapies for autoimmune disease. Nat. Rev. Rheumatol..

[8] Hu L., Liu R., Zhang L. (2022). Advance in bone destruction participated by JAK/STAT in rheumatoid arthritis and therapeutic effect of JAK/STAT inhibitors. Int. Immunopharmacol..

[9] Tuttle J., Drescher E., Simón-Campos Jesus A., Emery P., Greenwald M., Kivitz A., Rha H., Yachi P., Kiley C., Nirula A. (2023). A Phase 2 Trial of Peresolimab for Adults with Rheumatoid Arthritis. N. Engl. J. Med..

[10] Rivellese F., Surace A.E.A., Goldmann K., Sciacca E., Çubuk C., Giorli G., John C.R., Nerviani A., Fossati-Jimack L., Thorborn G. (2022). Rituximab versus tocilizumab in rheumatoid arthritis: Synovial biopsy-based biomarker analysis of the phase 4 R4RA randomized trial. Nat. Med..

[11] Cheema K.S., Bit Mansour A., Raychaudhuri S.P. (2025). What’s new on the horizon for rheumatoid arthritis management. Best Pract. Res. Clin. Rheumatol..

[12] Konzett V., Aletaha D. (2024). Management strategies in rheumatoid arthritis. Nat. Rev. Rheumatol..

[13] Singh J.A., Cameron C., Noorbaloochi S., Cullis T., Tucker M., Christensen R., Ghogomu E.T., Coyle D., Clifford T., Tugwell P. (2015). Risk of serious infection in biological treatment of patients with rheumatoid arthritis: A systematic review and meta-analysis. Lancet.

[14] Smolen J.S., Landewé R.B.M., Bijlsma J.W.J., Burmester G.R., Dougados M., Kerschbaumer A., McInnes I.B., Sepriano A., van Vollenhoven R.F., de Wit M. (2020). EULAR recommendations for the management of rheumatoid arthritis with synthetic and biological disease-modifying antirheumatic drugs: 2019 update. Ann. Rheum. Dis..

[15] Giollo A., Fuzzi E., Doria A. (2022). Methotrexate in early rheumatoid arthritis: Is the anchor drug still holding?. Autoimmun. Rev..

[16] Zhang M., Hu W., Cai C., Wu Y., Li J., Dong S. (2022). Advanced application of stimuli-responsive drug delivery system for inflammatory arthritis treatment. Mater. Today Bio.

[17] Fraenkel L., Bathon J.M., England B.R., St. Clair E.W., Arayssi T., Carandang K., Deane K.D., Genovese M., Huston K.K., Kerr G. (2021). 2021 American College of Rheumatology Guideline for the Treatment of Rheumatoid Arthritis. Arthritis Care Res..

[18] Ren S., Xu Y., Dong X., Mu Q., Chen X., Yu Y., Su G. (2024). Nanotechnology-empowered combination therapy for rheumatoid arthritis: Principles, strategies, and challenges. J. Nanobiotechnol..

[19] Guo Q., Wang Y., Xu D., Nossent J., Pavlos N.J., Xu J. (2018). Rheumatoid arthritis: Pathological mechanisms and modern pharmacologic therapies. Bone Res..

[20] van Venrooij W.J., van Beers J.J.B.C., Pruijn G.J.M. (2011). Anti-CCP antibodies: The past, the present and the future. Nat. Rev. Rheumatol..

[21] Aldridge J., Ekwall A.-K.H., Mark L., Bergström B., Andersson K., Gjertsson I., Lundell A.-C., Rudin A. (2020). T helper cells in synovial fluid of patients with rheumatoid arthritis primarily have a Th1 and a CXCR3^+^Th2 phenotype. Arthritis Res. Ther..

[22] Li B., Su R., Guo Q., Su R., Gao C., Li X., Wang C. (2024). Differential immunological profiles in seronegative versus seropositive rheumatoid arthritis: Th17/Treg dysregulation and IL-4. Front. Immunol..

[23] Firestein G.S., McInnes I.B. (2017). Immunopathogenesis of Rheumatoid Arthritis. Immunity.

[24] Rossetti M., Spreafico R., Consolaro A., Leong J.Y., Chua C., Massa M., Saidin S., Magni-Manzoni S., Arkachaisri T., Wallace C.A. (2017). TCR repertoire sequencing identifies synovial Treg cell clonotypes in the bloodstream during active inflammation in human arthritis. Ann. Rheum. Dis..

[25] Song W., Craft J. (2019). T follicular helper cell heterogeneity: Time, space, and function. Immunol. Rev..

[26] Wei X., Niu X. (2023). T follicular helper cells in autoimmune diseases. J. Autoimmun..

[27] Narasimhan P.B., Marcovecchio P., Hamers A.A.J., Hedrick C.C. (2019). Nonclassical Monocytes in Health and Disease. Annu. Rev. Immunol..

[28] Kurowska-Stolarska M., Alivernini S. (2022). Synovial tissue macrophages in joint homeostasis, rheumatoid arthritis and disease remission. Nat. Rev. Rheumatol..

[29] Nygaard G., Firestein G.S. (2020). Restoring synovial homeostasis in rheumatoid arthritis by targeting fibroblast-like synoviocytes. Nat. Rev. Rheumatol..

[30] Misharin A.V., Cuda C.M., Saber R., Turner J.D., Gierut A.K., Haines G.K., Berdnikovs S., Filer A., Clark A.R., Buckley C.D. (2014). Nonclassical Ly6C^−^ Monocytes Drive the Development of Inflammatory Arthritis in Mice. Cell Rep..

[31] Li M., Deng T., Chen Q., Jiang S., Li H., Li J., You S., Xie H.Q., Shen B. (2025). A versatile platform based on matrix metalloproteinase-sensitive peptides for novel diagnostic and therapeutic strategies in arthritis. Bioact. Mater..

[32] Hascoët E., Blanchard F., Blin-Wakkach C., Guicheux J., Lesclous P., Cloitre A. (2023). New insights into inflammatory osteoclast precursors as therapeutic targets for rheumatoid arthritis and periodontitis. Bone Res..

[33] Ah Kioon M.D., Laurent P., Chaudhary V., Du Y., Crow M.K., Barrat F.J. (2024). Modulation of plasmacytoid dendritic cells response in inflammation and autoimmunity. Immunol. Rev..

[34] Udalova I.A., Mantovani A., Feldmann M. (2016). Macrophage heterogeneity in the context of rheumatoid arthritis. Nat. Rev. Rheumatol..

[35] Suwa Y., Nagafuchi Y., Yamada S., Fujio K. (2023). The role of dendritic cells and their immunometabolism in rheumatoid arthritis. Front. Immunol..

[36] Morris G., Gevezova M., Sarafian V., Maes M. (2022). Redox regulation of the immune response. Cell. Mol. Immunol..

[37] Yang S., Zhao M., Jia S. (2023). Macrophage: Key player in the pathogenesis of autoimmune diseases. Front. Immunol..

[38] Raposo B., Klareskog L., Robinson W.H., Malmström V., Grönwall C. (2024). The peculiar features, diversity and impact of citrulline-reactive autoantibodies. Nat. Rev. Rheumatol..

[39] Alghazali T., Saleh R.O., Uthirapathy S., Ballal S., Abullais S.S., Kalia R., Arya R., Sharma R., Kumar A., Abdulamer R.S. (2025). Rheumatoid arthritis unmasked: The power of B cell depletion therapy. Mol. Biol. Rep..

[40] Pertsinidou E., Saevarsdottir S., Manivel V.A., Klareskog L., Alfredsson L., Mathsson-Alm L., Hansson M., Cornillet M., Serre G., Holmdahl R. (2024). In early rheumatoid arthritis, anticitrullinated peptide antibodies associate with low number of affected joints and rheumatoid factor associates with systemic inflammation. Ann. Rheum. Dis..

[41] Antonioli L., Fornai M., Pellegrini C., Masi S., Puxeddu I., Blandizzi C. (2020). Ectopic Lymphoid Organs and Immune-Mediated Diseases: Molecular Basis for Pharmacological Approaches. Trends Mol. Med..

[42] Cascão R., Rosário H.S., Souto-Carneiro M.M., Fonseca J.E. (2010). Neutrophils in rheumatoid arthritis: More than simple final effectors. Autoimmun. Rev..

[43] Fresneda Alarcon M., McLaren Z., Wright H.L. (2021). Neutrophils in the Pathogenesis of Rheumatoid Arthritis and Systemic Lupus Erythematosus: Same Foe Different M.O. Front. Immunol..

[44] Wright H.L., Lyon M., Chapman E.A., Moots R.J., Edwards S.W. (2020). Rheumatoid Arthritis Synovial Fluid Neutrophils Drive Inflammation Through Production of Chemokines, Reactive Oxygen Species, and Neutrophil Extracellular Traps. Front. Immunol..

[45] Frade-Sosa B., Sanmartí R. (2023). Neutrophils, neutrophil extracellular traps, and rheumatoid arthritis: An updated review for clinicians. Reumatol. Clin..

[46] Khandpur R., Carmona-Rivera C., Vivekanandan-Giri A., Gizinski A., Yalavarthi S., Knight J.S., Friday S., Li S., Patel R.M., Subramanian V. (2013). NETs Are a Source of Citrullinated Autoantigens and Stimulate Inflammatory Responses in Rheumatoid Arthritis. Sci. Transl. Med..

[47] Tang J., Xia J., Gao H., Jiang R., Xiao L., Sheng H., Lin J. (2024). IL33-induced neutrophil extracellular traps (NETs) mediate a positive feedback loop for synovial inflammation and NET amplification in rheumatoid arthritis. Exp. Mol. Med..

[48] Coutant F. (2019). Pathogenic effects of anti-citrullinated protein antibodies in rheumatoid arthritis—Role for glycosylation. Jt. Bone Spine.

[49] Lebre M.C., Jongbloed S.L., Tas S.W., Smeets T.J., McInnes I.B., Tak P.P. (2008). Rheumatoid arthritis synovium contains two subsets of CD83^−^DC-LAMP^−^ dendritic cells with distinct cytokine profiles. Am. J. Pathol..

[50] Jongbloed S.L., Lebre M.C., Fraser A.R., Gracie J.A., Sturrock R.D., Tak P.P., McInnes I.B. (2006). Enumeration and phenotypical analysis of distinct dendritic cell subsets in psoriatic arthritis and rheumatoid arthritis. Arthritis Res. Ther..

[51] Page G., Miossec P. (2004). Paired synovium and lymph nodes from rheumatoid arthritis patients differ in dendritic cell and chemokine expression. J. Pathol..

[52] Bottini N., Firestein G.S. (2013). Duality of fibroblast-like synoviocytes in RA: Passive responders and imprinted aggressors. Nat. Rev. Rheumatol..

[53] Komatsu N., Takayanagi H. (2022). Mechanisms of joint destruction in rheumatoid arthritis-immune cell–fibroblast–bone interactions. Nat. Rev. Rheumatol..

[54] Karouzakis E., Raza K., Kolling C., Buckley C.D., Gay S., Filer A., Ospelt C. (2018). Analysis of early changes in DNA methylation in synovial fibroblasts of RA patients before diagnosis. Sci. Rep..

[55] Tsaltskan V., Firestein G.S. (2022). Targeting fibroblast-like synoviocytes in rheumatoid arthritis. Curr. Opin. Pharmacol..

[56] Liu X., Li J., Sun L., Wang T., Liang W. (2023). The association between neutrophil-to-lymphocyte ratio and disease activity in rheumatoid arthritis. Inflammopharmacology.

[57] Mousavi M.J., Karami J., Aslani S., Tahmasebi M.N., Vaziri A.S., Jamshidi A., Farhadi E., Mahmoudi M. (2021). Transformation of fibroblast-like synoviocytes in rheumatoid arthritis; from a friend to foe. Autoimmun. Highlights.

[58] Rufino A.T., Freitas M., Proença C., Ferreira de Oliveira J.M.P., Fernandes E., Ribeiro D. (2024). Rheumatoid arthritis molecular targets and their importance to flavonoid-based therapy. Med. Res. Rev..

[59] Wang Y., Wu H., Deng R. (2021). Angiogenesis as a potential treatment strategy for rheumatoid arthritis. Eur. J. Pharmacol..

[60] Veale D.J., Orr C., Fearon U. (2017). Cellular and molecular perspectives in rheumatoid arthritis. Semin. Immunopathol..

[61] Fearon U., Canavan M., Biniecka M., Veale D.J. (2016). Hypoxia, mitochondrial dysfunction and synovial invasiveness in rheumatoid arthritis. Nat. Rev. Rheumatol..

[62] Fearon U., Hanlon M.M., Floudas A., Veale D.J. (2022). Cellular metabolic adaptations in rheumatoid arthritis and their therapeutic implications. Nat. Rev. Rheumatol..

[63] Henry Ó.C., O’Neill L.A.J. (2025). Metabolic Reprogramming in Stromal and Immune Cells in Rheumatoid Arthritis and Osteoarthritis: Therapeutic Possibilities. Eur. J. Immunol..

[64] Wang X., Fan D., Cao X., Ye Q., Wang Q., Zhang M., Xiao C. (2022). The Role of Reactive Oxygen Species in the Rheumatoid Arthritis-Associated Synovial Microenvironment. Antioxidants.

[65] Kardeş S., Karagülle M., Durak İ., Avcı A., Karagülle M.Z. (2018). Association of oxidative stress with clinical characteristics in patients with rheumatoid arthritis. Eur. J. Clin. Investig..

[66] Guo Q., Jin Y., Chen X., Ye X., Shen X., Lin M., Zeng C., Zhou T., Zhang J. (2024). NF-κB in biology and targeted therapy: New insights and translational implications. Signal Transduct. Target. Ther..

[67] Lefèvre S., Knedla A., Tennie C., Kampmann A., Wunrau C., Dinser R., Korb A., Schnäker E.M., Tarner I.H., Robbins P.D. (2009). Synovial fibroblasts spread rheumatoid arthritis to unaffected joints. Nat. Med..

[68] Trejo-Zambrano M.I., Gómez-Bañuelos E., Andrade F. (2022). Redox-Mediated Carbamylation As a Hapten Model Applied to the Origin of Antibodies to Modified Proteins in Rheumatoid Arthritis. Antioxid. Redox Signal..

[69] Makkar R., Sehgal A., Singh S., Sharma N., Rawat R., Rashid S., Vargas-De-La-Cruz C., Yadav S., Bungau S.G., Behl T. (2023). Current trends in epigenetic, cellular and molecular pathways in management of rheumatoid arthritis. Inflammopharmacology.

[70] Samuels J.S., Holland L., López M., Meyers K., Cumbie W.G., McClain A., Ignatowicz A., Nelson D., Shashidharamurthy R. (2018). Prostaglandin E2 and IL-23 interconnects STAT3 and RoRγ pathways to initiate Th17 CD4^+^ T-cell development during rheumatoid arthritis. Inflamm. Res..

[71] Koper-Lenkiewicz O.M., Sutkowska K., Wawrusiewicz-Kurylonek N., Kowalewska E., Matowicka-Karna J. (2022). Proinflammatory Cytokines (IL-1, -6, -8, -15, -17, -18, -23, TNF-α) Single Nucleotide Polymorphisms in Rheumatoid Arthritis-A Literature Review. Int. J. Mol. Sci..

[72] Dong P., Qiu H., Wen R., Zou X., Sun X., Yu L., Zhang S., Wu Y., Lan F. (2024). Reactive Oxygen and Nitrogen Species—“Nanosweeper” for Rheumatoid Arthritis Theranostics by Macrophage Reprogramming. ACS Appl. Mater. Interfaces.

[73] Minhas R., Bansal Y., Bansal G. (2020). Inducible nitric oxide synthase inhibitors: A comprehensive update. Med. Res. Rev..

[74] Oza P.P., Kashfi K. (2023). The Triple Crown: NO, CO, and H_2_S in cancer cell biology. Pharmacol. Ther..

[75] Tripodi G., Lombardo M., Kerav S., Aiello G., Baldelli S. (2025). Nitric Oxide in Parkinson’s Disease: The Potential Role of Dietary Nitrate in Enhancing Cognitive and Motor Health via the Nitrate-Nitrite-Nitric Oxide Pathway. Nutrients.

[76] Wierońska J.M., Cieślik P., Kalinowski L. (2021). Nitric Oxide-Dependent Pathways as Critical Factors in the Consequences and Recovery after Brain Ischemic Hypoxia. Biomolecules.

[77] Sellam J., Berenbaum F. (2010). The role of synovitis in pathophysiology and clinical symptoms of osteoarthritis. Nat. Rev. Rheumatol..

[78] Steinmetz-Späh J., Jakobsson P.J. (2023). The anti-inflammatory and vasoprotective properties of mPGES-1 inhibition offer promising therapeutic potential. Expert Opin. Ther. Targets.

[79] Jia X.Y., Chang Y., Sun X.J., Dai X., Wei W. (2014). The role of prostaglandin E2 receptor signaling of dendritic cells in rheumatoid arthritis. Int. Immunopharmacol..

[80] Park J.Y., Pillinger M.H., Abramson S.B. (2006). Prostaglandin E2 synthesis and secretion: The role of PGE2 synthases. Clin. Immunol..

[81] Wang P., You X., Yan Y., Singh G.K., Li X., Zhou W., Liu W., Zhang F., Lv Y., Yang L. (2011). Cyclic mechanical stretch downregulates IL-1β-induced COX-2 expression and PGE_2_ production in rheumatoid arthritis fibroblast-like synoviocytes. Connect. Tissue Res..

[82] Ching M.M., Reader J., Fulton A.M. (2020). Eicosanoids in Cancer: Prostaglandin E_2_ Receptor 4 in Cancer Therapeutics and Immunotherapy. Front. Pharmacol..

[83] Wehbi V.L., Taskén K. (2016). Molecular Mechanisms for cAMP-Mediated Immunoregulation in T cells—Role of Anchored Protein Kinase A Signaling Units. Front. Immunol..

[84] Deane K.D., Holers V.M., Emery P., Mankia K., El-Gabalawy H., Sparks J.A., Costenbader K.H., Schett G., van der Helm-van Mil A., van Schaardenburg D. (2025). Therapeutic interception in individuals at risk of rheumatoid arthritis to prevent clinically impactful disease. Ann. Rheum. Dis..

[85] de Brito Rocha S., Baldo D.C., Andrade L.E.C. (2019). Clinical and pathophysiologic relevance of autoantibodies in rheumatoid arthritis. Adv. Rheumatol..

[86] Volkov M., van Schie K.A., van der Woude D. (2020). Autoantibodies and B Cells: The ABC of rheumatoid arthritis pathophysiology. Immunol. Rev..

[87] Loucks A., Maerz T., Hankenson K., Moeser A., Colbath A. (2023). The multifaceted role of mast cells in joint inflammation and arthritis. Osteoarthr. Cartil..

[88] Coss S.L., Zhou D., Chua G.T., Aziz R.A., Hoffman R.P., Wu Y.L., Ardoin S.P., Atkinson J.P., Yu C.Y. (2023). The complement system and human autoimmune diseases. J. Autoimmun..

[89] Hysa E., Cutolo C.A., Gotelli E., Paolino S., Cimmino M.A., Pacini G., Pizzorni C., Sulli A., Smith V., Cutolo M. (2021). Ocular microvascular damage in autoimmune rheumatic diseases: The pathophysiological role of the immune system. Autoimmun. Rev..

[90] Sung W.Y., Tsai W.C. (2021). Rethink About the Role of Rheumatoid Factor and Anti-citrullinated Protein Antibody in Rheumatoid Arthritis. Rheumatol. Immunol. Res..

[91] Luo L., Chen H., Xie K., Xiang J., Chen J., Lin Z. (2024). Cathepsin B serves as a potential prognostic biomarker and correlates with ferroptosis in rheumatoid arthritis. Int. Immunopharmacol..

[92] Clanchy F.I.L., Borghese F., Bystrom J., Balog A., Penn H., Taylor P.C., Stone T.W., Mageed R.A., Williams R.O. (2022). Disease status in human and experimental arthritis, and response to TNF blockade, is associated with MHC class II invariant chain (CD74) isoform expression. J. Autoimmun..

[93] Shokry A.A., El-Shiekh R.A., Kamel G., Bakr A.F., Sabry D., Ramadan A. (2022). Anti-arthritic activity of the flavonoids fraction of ivy leaves (*Hedera helix* L.) standardized extract in adjuvant induced arthritis model in rats in relation to its metabolite profile using LC/MS. Biomed. Pharmacother..

[94] Zhang W., Yin G., Zhao H., Ling H., Xie Z., Xiao C., Chen Y., Lin Y., Jiang T., Jin S. (2021). Secreted KIAA1199 promotes the progression of rheumatoid arthritis by mediating hyaluronic acid degradation in an ANXA1-dependent manner. Cell Death Dis..

[95] Agnihotri P., Saquib M., Joshi L., Malik S., Chakraborty D., Sarkar A., Kumar U., Biswas S. (2024). Integrative metabolomic-proteomic analysis uncovers a new therapeutic approach in targeting rheumatoid arthritis. Arthritis Res. Ther..

[96] Meng X., Chen Z., Li T., Nie Z., Han H., Zhong S., Yin Z., Sun S., Xie J., Shen J. (2024). Role and Therapeutic Potential for Targeting Fibroblast Growth Factor 10/FGFR1 in Relapsed Rheumatoid Arthritis. Arthritis Rheumatol..

[97] Fang Q., Zhou C., Nandakumar K.S. (2020). Molecular and Cellular Pathways Contributing to Joint Damage in Rheumatoid Arthritis. Mediat. Inflamm..

[98] Liu S., Ma H., Zhang H., Deng C., Xin P. (2021). Recent advances on signaling pathways and their inhibitors in rheumatoid arthritis. Clin. Immunol..

[99] Noort A.R., Tak P.P., Tas S.W. (2015). Non-canonical NF-κB signaling in rheumatoid arthritis: Dr Jekyll and Mr Hyde?. Arthritis Res. Ther..

[100] Ciobanu D.A., Poenariu I.S., Crînguș L.I., Vreju F.A., Turcu-Stiolica A., Tica A.A., Padureanu V., Dumitrascu R.M., Banicioiu-Covei S., Dinescu S.C. (2020). JAK/STAT pathway in pathology of rheumatoid arthritis (Review). Exp. Ther. Med..

[101] Delgado-Pujol E.J., Martínez G., Casado-Jurado D., Vázquez J., León-Barberena J., Rodríguez-Lucena D., Torres Y., Alcudia A., Begines B. (2025). Hydrogels and Nanogels: Pioneering the Future of Advanced Drug Delivery Systems. Pharmaceutics.

[102] Cao H., Duan L., Zhang Y., Cao J., Zhang K. (2021). Current hydrogel advances in physicochemical and biological response-driven biomedical application diversity. Signal Transduct. Target. Ther..

[103] Hu K., Xiao M., Chen S., Huang Y., Hou Z., Li X., Yang L. (2025). Innovative applications of natural polysaccharide polymers in intravesical therapy of bladder diseases. Carbohydr. Polym..

[104] Asadi K., Samiraninezhad N., Akbarizadeh A.R., Amini A., Gholami A. (2024). Stimuli-responsive hydrogel based on natural polymers for breast cancer. Front. Chem..

[105] Ho T.C., Chang C.C., Chan H.P., Chung T.W., Shu C.W., Chuang K.P., Duh T.H., Yang M.H., Tyan Y.C. (2022). Hydrogels: Properties and Applications in Biomedicine. Molecules.

[106] Khan F., Atif M., Haseen M., Kamal S., Khan M.S., Shahid S., Nami S.A.A. (2022). Synthesis, classification and properties of hydrogels: Their applications in drug delivery and agriculture. J. Mater. Chem. B.

[107] Soliman B.G., Nguyen A.K., Gooding J.J., Kilian K.A. (2024). Advancing Synthetic Hydrogels through Nature-Inspired Materials Chemistry. Adv. Mater..

[108] Liu S., Han F., Chen P., Zhang R., Tao Y. (2025). Injectable and drug-loaded gelatin methacrylate and carboxymethylated-sulfated xanthan gum hydrogels as biomimetic mineralization constructs. Carbohydr. Polym..

[109] Tang J., Cheng X., Pan R., Li J., Li Z., Liang W., Xie H., Zhang H., Zhao J., Yu K. (2025). Polyvinyl pyrrolidone/carboxymethyl chitosan hydrogel loaded with *Paris polyphylla* var. *yunnanensis* extracellular vesicles promotes wound healing. Int. J. Biol. Macromol..

[110] Yao P., Tan Z., Weng B., Wang X., Wang H., Yang G., Sun F., Zhao Y. (2024). Locally Injectable Chitosan/β-Glycerophosphate Hydrogel Doped with Triptolide-Human Serum Albumin Nanoparticles for Treating Rheumatoid Arthritis. Pharmaceuticals.

[111] Chen J., Luo J., Feng J., Wang Y., Lv H., Zhou Y. (2024). Spatiotemporal controlled released hydrogels for multi-system regulated bone regeneration. J. Control. Release.

[112] Chen J., Wang H., Long F., Bai S., Wang Y. (2023). Dynamic supramolecular hydrogels mediated by chemical reactions. Chem. Commun..

[113] Ren H., Zhang Z., Cheng X., Zou Z., Chen X., He C. (2023). Injectable, self-healing hydrogel adhesives with firm tissue adhesion and on-demand biodegradation for sutureless wound closure. Sci. Adv..

[114] Wu Q., Yang R., Fan W., Wang L., Zhan J., Cao T., Liu Q., Piao X., Zhong Y., Zhao W. (2024). Spermidine-Functionalized Injectable Hydrogel Reduces Inflammation and Enhances Healing of Acute and Diabetic Wounds In Situ. Adv. Sci..

[115] Liu L., Wang W., Huang L., Xian Y., Ma W., Fan J., Li Y., Liu H., Zheng Z., Wu D. (2024). Injectable pathological microenvironment-responsive anti-inflammatory hydrogels for ameliorating intervertebral disc degeneration. Biomaterials.

[116] Gajendiran M., Rhee J.S., Kim K. (2018). Recent Developments in Thiolated Polymeric Hydrogels for Tissue Engineering Applications. Tissue Eng. Part B Rev..

[117] Wang Q., Cheng Q., Yao G., Wang Z., Zhu L., Zeng Z., Jia L., Du Y., Xue J., Gao C. (2024). A cationic hydrogel with anti-IL-17A-specific nanobodies for rheumatoid arthritis treatment via inhibition of inflammatory activities of neutrophils. Nano Today.

[118] Rovers M.M., Rogkoti T., Bakker B.K., Bakal K.J., van Genderen M.H.P., Salmeron-Sanchez M., Dankers P.Y.W. (2024). Using a Supramolecular Monomer Formulation Approach to Engineer Modular, Dynamic Microgels, and Composite Macrogels. Adv. Mater..

[119] Tolabi H., Davari N., Khajehmohammadi M., Malektaj H., Nazemi K., Vahedi S., Ghalandari B., Reis R.L., Ghorbani F., Oliveira J.M. (2023). Progress of Microfluidic Hydrogel-Based Scaffolds and Organ-on-Chips for the Cartilage Tissue Engineering. Adv. Mater..

[120] Qi X., Qin X., Yang R., Qin J., Li W., Luan K., Wu Z., Song L. (2016). Intra-articular Administration of Chitosan Thermosensitive In Situ Hydrogels Combined with Diclofenac Sodium-Loaded Alginate Microspheres. J. Pharm. Sci..

[121] Wu Y., Ge Y., Wang Z., Zhu Y., Tian T., Wei J., Jin Y., Zhao Y., Jia Q., Wu J. (2024). Synovium microenvironment-responsive injectable hydrogel inducing modulation of macrophages and elimination of synovial fibroblasts for enhanced treatment of rheumatoid arthritis. J. Nanobiotechnol..

[122] Singh R., Jadhav K., Kamboj R., Malhotra H., Ray E., Jhilta A., Dhir V., Verma R.K. (2024). Self-actuating inflammation responsive hydrogel microsphere formulation for controlled drug release in rheumatoid arthritis (RA): Animal trials and study in human fibroblast like synoviocytes (hFLS) of RA patients. Biomater. Adv..

[123] Terriac L., Helesbeux J.J., Maugars Y., Guicheux J., Tibbitt M.W., Delplace V. (2024). Boronate Ester Hydrogels for Biomedical Applications: Challenges and Opportunities. Chem. Mater..

[124] Zhai Z., Dong X., Qi H., Tao R., Zhang P. (2023). Carbon Quantum Dots with High Photothermal Conversion Efficiency and Their Application in Photothermal Modulated Reversible Deformation of Poly(*N*-isopropylacrylamide) Hydrogel. ACS Appl. Bio Mater..

[125] Kikani T., Dave S., Thakore S. (2023). Functionalization of hyaluronic acid for development of self-healing hydrogels for biomedical applications: A review. Int. J. Biol. Macromol..

[126] Ahmed Y.W., Loukanov A., Tsai H.-C. (2024). State-of-the-Art Synthesis of Porous Polymer Materials and Their Several Fantastic Biomedical Applications: A Review. Adv. Healthc. Mater..

[127] Wang S., Liu Y., Sun Q., Zeng B., Liu C., Gong L., Wu H., Chen L., Jin M., Guo J. (2023). Triple Cross-linked Dynamic Responsive Hydrogel Loaded with Selenium Nanoparticles for Modulating the Inflammatory Microenvironment via PI3K/Akt/NF-κB and MAPK Signaling Pathways. Adv. Sci..

[128] Zhao G., Ren R., Wei X., Jia Z., Chen N., Sun Y., Zhao Z., Lele S.M., Zhong H.A., Goldring M.B. (2021). Thermoresponsive polymeric dexamethasone prodrug for arthritis pain. J. Control. Release.

[129] Du X., Lin Y., Shuai Z., Duan J., Wang C., Liu J., Jiang J., Wu J., Zhou M., Zhang Z. (2023). Nanocomposite hydrogel to deliver the immunomodulator lenalidomide and anti-inflammatory hesperidin locally to joints affected by rheumatoid arthritis. Chem. Eng. J..

[130] Wu Y., Wang Z., Ge Y., Zhu Y., Tian T., Wei J., Jin Y., Zhao Y., Jia Q., Wu J. (2024). Microenvironment Responsive Hydrogel Exerting Inhibition of Cascade Immune Activation and Elimination of Synovial Fibroblasts for Rheumatoid Arthritis Therapy. J. Control. Release.

[131] Xiong J., Xie R., Wang Y., Wang C., Ai Y., Zheng W., Ding M., Gao J., Wang J., Liang Q. (2020). Nitrite-Responsive Hydrogel: Smart Drug Release Depending on the Severity of the Nitric Oxide-Related Disease. ACS Appl. Mater. Interfaces.

[132] Xu M., Fu T., Zhang C., An Z., Yan J., Lu Z., Wu H., Liu J., Qiu L., Shi L. (2024). Prolonged, staged, and self-regulated methotrexate release coupled with ROS scavenging in an injectable hydrogel for rheumatoid arthritis therapy. J. Control. Release.

[133] Yu H., Gao R., Liu Y., Fu L., Zhou J., Li L. (2024). Stimulus-Responsive Hydrogels as Drug Delivery Systems for Inflammation Targeted Therapy. Adv. Sci..

[134] Liu X., Zhang Q., Cao Y., Hussain Z., Xu M., Liu Y., Ullah I., Lu Z., Osaka A., Lin J. (2024). An Injectable Hydrogel Composing Anti-Inflammatory and Osteogenic Therapy toward Bone Erosions Microenvironment Remodeling in Rheumatoid Arthritis. Adv. Healthc. Mater..

[135] Wang N., Ma J., Song W., Zhao C. (2023). An injectable hydrogel to disrupt neutrophil extracellular traps for treating rheumatoid arthritis. Drug Deliv..

[136] Yang R., Yan L., Xu T., Zhang K., Lu X., Xie C., Fu W. (2024). Injectable bioadhesive hydrogel as a local nanomedicine depot for targeted regulation of inflammation and ferroptosis in rheumatoid arthritis. Biomaterials.

[137] Lee S., Seo J., Kim Y.H., Ju H.J., Kim S., Ji Y.B., Lee H.B., Kim H.S., Choi S., Kim M.S. (2023). Enhanced intra-articular therapy for rheumatoid arthritis using click-crosslinked hyaluronic acid hydrogels loaded with toll-like receptor antagonizing peptides. Acta Biomater..

[138] Park J.H., Park S.H., Lee H.Y., Lee J.W., Lee B.K., Lee B.Y., Kim J.H., Kim M.S. (2018). An injectable, electrostatically interacting drug depot for the treatment of rheumatoid arthritis. Biomaterials.

[139] Li C., Du Y., Lv H., Zhang J., Zhuang P., Yang W., Zhang Y., Wang J., Cui W., Chen W. (2022). Injectable Amphipathic Artesunate Prodrug-Hydrogel Microsphere as Gene/Drug Nano-Microplex for Rheumatoid Arthritis Therapy. Adv. Funct. Mater..

[140] Zhao Y., Song S., Wang D., Liu H., Zhang J., Li Z., Wang J., Ren X., Zhao Y. (2022). Nanozyme-reinforced hydrogel as a H_2_O_2_-driven oxygenerator for enhancing prosthetic interface osseointegration in rheumatoid arthritis therapy. Nat. Commun..

[141] Geng W., Zhao J., Tao B., Yang Y., Duan Q., Gao P., He T., Liu S., Feng Q., Zhao P. (2024). Regulation of rheumatoid arthritis microenvironment via a self-healing injectable hydrogel for improved inflammation elimination and bone repair. Bioact. Mater..

[142] Zhu H., Wu X., Liu R., Zhao Y., Sun L. (2023). ECM-Inspired Hydrogels with ADSCs Encapsulation for Rheumatoid Arthritis Treatment. Adv. Sci..

[143] Liao H., Qi W., Xue Z., Wu K., Jiang L., Wu C., Huang Z., Li Q., Lu Y. (2023). A multifunctional supramolecular hydrogel that rapidly binds TNF-α for efficient reduction of synovial inflammation and cartilage destruction in rheumatoid arthritis. Chem. Eng. J..

[144] Zhou C., Hao W., Yao J., Zhu T., Sun M., Lu Y., Wang L., Zhou X., Loh J.L.C. (2025). Anti-inflammatory supramolecular hydrogel loaded chicoric acid based on graphene oxide modified hyaluronic acid and polyethylene glycol for rheumatoid arthritis treatment. Int. J. Biol. Macromol..

[145] Song Y., Yang P., Guo W., Lu P., Huang C., Cai Z., Jiang X., Yang G., Du Y., Zhao F. (2024). Supramolecular Hydrogel Dexamethasone-Diclofenac for the Treatment of Rheumatoid Arthritis. Nanomaterials.

[146] Kim K.S., Park S.J., Yang J.A., Jeon J.H., Bhang S.H., Kim B.S., Hahn S.K. (2011). Injectable hyaluronic acid-tyramine hydrogels for the treatment of rheumatoid arthritis. Acta Biomater..

[147] Li C., Liu R., Song Y., Chen Y., Zhu D., Yu L., Huang Q., Zhang Z., Xue Z., Hua Z. (2022). Hyaluronic Acid Hydrogels Hybridized with Au-Triptolide Nanoparticles for Intraarticular Targeted Multi-Therapy of Rheumatoid Arthritis. Front. Pharmacol..

[148] Ma Z., Tao C., Sun L., Qi S., Le Y., Wang J., Li C., Liu X., Zhang J., Zhao J. (2019). In Situ Forming Injectable Hydrogel for Encapsulation of Nanoiguratimod and Sustained Release of Therapeutics. Int. J. Nanomed..

[149] Rui K., Tang X., Shen Z., Jiang C., Zhu Q., Liu S., Che N., Tian J., Ling J., Yang Y. (2023). Exosome inspired photo-triggered gelation hydrogel composite on modulating immune pathogenesis for treating rheumatoid arthritis. J. Nanobiotechnol..

[150] Shi G., Zhou Y., Liu W., Chen C., Wei Y., Yan X., Wu L., Wang W., Sun L., Zhang T. (2023). Bone-derived MSCs encapsulated in alginate hydrogel prevent collagen-induced arthritis in mice through the activation of adenosine A(2A/2B) receptors in tolerogenic dendritic cells. Acta Pharm. Sin. B.

[151] Wang F., Han Y., Zhou Q., Sheng S., Hu Y., Zhang H., Chen X., He C., Tan H., Bai L. (2025). Polymer-modified DNA hydrogels for living mitochondria and nanozyme delivery in the treatment of rheumatoid arthritis. Bioact. Mater..

[152] Zhao Y., Gao C., Liu H., Liu H., Feng Y., Li Z., Liu H., Wang J., Yang B., Lin Q. (2021). Infliximab-based self-healing hydrogel composite scaffold enhances stem cell survival, engraftment, and function in rheumatoid arthritis treatment. Acta Biomater..

[153] Chiang C.-W., Hsiao Y.-C., Jheng P.-R., Chen C.-H., Manga Y.B., Lekha R., Chao K.-M., Ho Y.-C., Chuang E.-Y. (2021). Strontium ranelate-laden near-infrared photothermal-inspired methylcellulose hydrogel for arthritis treatment. Mater. Sci. Eng. C.

[154] Ali A., Jori C., Kanika, Kumar A., Vyawahare A., Kumar J., Kumar B., Ahmad A., Fareed M., Ali N. (2024). A bioactive and biodegradable vitamin C stearate-based injectable hydrogel alleviates experimental inflammatory arthritis. Biomater. Sci..

[155] Joshi N., Yan J., Levy S., Bhagchandani S., Slaughter K.V., Sherman N.E., Amirault J., Wang Y., Riegel L., He X. (2018). Towards an arthritis flare-responsive drug delivery system. Nat. Commun..

[156] Wang K., Yin C., Ye X., Chen Q., Wu J., Chen Y., Li Y., Wang J., Duan C., Lu A. (2023). A Metabolic Driven Bio-Responsive Hydrogel Loading Psoralen for Therapy of Rheumatoid Arthritis. Small.

[157] Kim T., Suh J., Kim W.J. (2021). Polymeric Aggregate-Embodied Hybrid Nitric-Oxide-Scavenging and Sequential Drug-Releasing Hydrogel for Combinatorial Treatment of Rheumatoid Arthritis. Adv. Mater..

[158] Yeo J., Lee Y.M., Lee J., Park D., Kim K., Kim J., Park J., Kim W.J. (2019). Nitric Oxide-Scavenging Nanogel for Treating Rheumatoid Arthritis. Nano Lett..

[159] Liu L., Deng D., Li C., Huang G., Zhang W., Liang T., Liang R., Liang M., Su Y., Lin C. (2024). The combination of modified acupuncture needle and melittin hydrogel as a novel therapeutic approach for rheumatoid arthritis treatment. J. Nanobiotechnol..

[160] Liu X., Chen X., Fei Y., Zhang J., Yue O., Wang X., Jiang H. (2025). Locally Injectable, ROS-Scavenging, and ROS-/pH-Responsive Polymeric-Micelles-Embedded Hydrogels for Precise Minimally Invasive and Long-Lasting Rheumatoid Therapy. Adv. Healthc. Mater..

[161] Wang Q.S., Xu B.X., Fan K.J., Li Y.W., Wu J., Wang T.Y. (2020). Dexamethasone-Loaded Thermosensitive Hydrogel Suppresses Inflammation and Pain in Collagen-Induced Arthritis Rats. Drug Des. Dev. Ther..

[162] Pan W., Dai C., Li Y., Yin Y., Gong L., Machuki J.O., Yang Y., Qiu S., Guo K., Gao F. (2020). PRP-chitosan thermoresponsive hydrogel combined with black phosphorus nanosheets as injectable biomaterial for biotherapy and phototherapy treatment of rheumatoid arthritis. Biomaterials.

[163] Lee S., Choi S., Kim M.S. (2024). Intra-articular hydrogel formulation prolongs the in vivo stability of Toll-like receptor antagonistic peptides for rheumatoid arthritis treatment. J. Control. Release.

[164] Xu L., Qin J., Ma X., Wang Q., Wu W., Huang H., Cai L. (2024). Chitosan-based self-healing thermosensitive hydrogel loaded with siHMGB1 for treatment of rheumatoid arthritis via macrophage repolarization. Int. J. Biol. Macromol..

[165] Gong H., Hua Y., Wang Y., Zhang X., Wang H., Zhao Z., Zhang Y. (2024). Fabrication of a novel macrophage-targeted biomimetic delivery composite hydrogel with multiple-sensitive properties for tri-modal combination therapy of rheumatoid arthritis. Int. J. Pharm..

[166] Makled S., Abbas H., Ali M.E., Zewail M. (2024). Melatonin hyalurosomes in collagen thermosensitive gel as a potential repurposing approach for rheumatoid arthritis management via the intra-articular route. Int. J. Pharm..

[167] Rui X., Yang Y., Chen Q., Wu J., Chen J., Zhang Q., Ren R., Yin D. (2020). Imperative and effective reversion of synovial hyperplasia and cartilage destruction in rheumatoid arthritis through multiple synergistic effects of O^2^ and Ca^2+^. Mater. Sci. Eng. C Mater. Biol. Appl..

[168] Chen W., Li Z., Wang Z., Gao H., Ding J., He Z. (2020). Intraarticular Injection of Infliximab-Loaded Thermosensitive Hydrogel Alleviates Pain and Protects Cartilage in Rheumatoid Arthritis. J. Pain Res..

[169] Wang T., Huang C., Fang Z., Bahatibieke A., Fan D., Wang X., Zhao H., Xie Y., Qiao K., Xiao C. (2024). A dual dynamically cross-linked hydrogel promotes rheumatoid arthritis repair through ROS initiative regulation and microenvironment modulation-independent triptolide release. Mater. Today Bio.

[170] Bahatibieke A., Zhao J., Fan D., Zhou Z., Li J., Wang X., Zhao H., Wang T., Fang Z., Xie Y. (2025). Sea-Island Micelle Structured Hydrogel Scaffold: A Dual-Action Approach to Combat Cartilage Damage under RA Conditions. ACS Appl. Mater. Interfaces.

[171] Yin N., Guo X., Sun R., Liu H., Tang L., Gou J., Yin T., He H., Zhang Y., Tang X. (2020). Intra-articular injection of indomethacin-methotrexate in situ hydrogel for the synergistic treatment of rheumatoid arthritis. J. Mater. Chem. B.

[172] Du Y., Li C., Zhang Y., Xiong W., Wang F., Wang J., Zhang Y., Deng L., Li X., Chen W. (2022). In Situ-Activated Phospholipid-Mimic Artemisinin Prodrug via Injectable Hydrogel Nano/Microsphere for Rheumatoid Arthritis Therapy. Research.

[173] Li Y., Zhu H., Liu R., Zhao Y., Sun L. (2024). Hierarchical Microcarriers Loaded with Peptide Dendrimer-Grafted Methotrexate for Rheumatoid Arthritis Treatment. Small Sci..

[174] Abou-ElNour M., Soliman M.E., Skouras A., Casettari L., Geneidi A.S., Ishak R.A.H. (2020). Microparticles-in-Thermoresponsive/Bioadhesive Hydrogels as a Novel Integrated Platform for Effective Intra-articular Delivery of Triamcinolone Acetonide. Mol. Pharm..

[175] Xu C., Wang S., Wang H., Liu K., Zhang S., Chen B., Liu H., Tong F., Peng F., Tu Y. (2021). Magnesium-Based Micromotors as Hydrogen Generators for Precise Rheumatoid Arthritis Therapy. Nano Lett..

[176] Wu L., Zhao K., Xu L., Cui J., Ruan L., Bei S., Cao J., Qi X., Shen S. (2024). Macrophages-mediated delivery of protoporphyrin for sonodynamic therapy of rheumatoid arthritis. Ultrason. Sonochem.

[177] Li W., Song Y., Liang X., Zhou Y., Xu M., Lu Q., Wang X., Li N. (2021). Mutual-reinforcing sonodynamic therapy against Rheumatoid Arthritis based on sparfloxacin sonosensitizer doped concave-cubic rhodium nanozyme. Biomaterials.

[178] Elshabrawy H.A., Abo Dena A.S., El-Sherbiny I.M. (2024). Triple-layered platform utilizing electrospun nanofibers and 3D-printed sodium alginate-based hydrogel for effective topical treatment of rheumatoid arthritis. Int. J. Biol. Macromol..

[179] Clegg J.R., Adebowale K., Zhao Z., Mitragotri S. (2024). Hydrogels in the clinic: An update. Bioeng. Transl. Med..

[180] Fleischmann R., Furst D.E. (2020). Safety of repository corticotropin injection as an adjunctive therapy for the treatment of rheumatoid arthritis. Expert Opin. Drug Saf..

